# Third-generation RNA Amplicon Sequencing Reveals the Dynamics of Microbial Communities in the Tidal Reach of a Subtropical Estuary

**DOI:** 10.1007/s00248-026-02804-4

**Published:** 2026-06-23

**Authors:** Qiqi Deng, Jialing Li, Xiaoqing Luo, Ziqi Peng, Wendong Xian, Haixian Xiong, Leiping Ye, Huan Liu, Jie Ren, Jiaxue Wu, Wenjun Li, Pandeng Wang

**Affiliations:** 1 State Key Laboratory of Biocontrol, Guangdong Provincial Key Laboratory of Plant Stress Biology, School of Life Sciences, Sun Yat-Sen University, Guangzhou, 510275 China; 2 Southern Marine Science and Engineering Guangdong Laboratory (Guangzhou), Guangzhou, 511458 China; 3 Marine Science and Technology College, Zhejiang Ocean University, Zhoushan, 316000 China; 4 School of Marine Sciences, Southern Marine Science and Engineering Guangdong Laboratory (Zhuhai), Sun Yat-sen University, Zhuhai, 519082, China; 5 School of Pharmacy, Shenzhen University Medical School, Shenzhen University, Shenzhen, 518055 China

**Keywords:** Salinity, Microbial diversity, Co-occurrence network, Tidal reach

## Abstract

Estuarine ecosystems are characterized by periodic tidal cycles that induce dynamic salinity fluctuations, yet how these dynamic salinity changes affect microbial diversity, community composition, and network structure remains poorly understood. Answering this question would enhance our understanding of the effects of periodic tides on microbial communities in estuaries. To address this knowledge gap, we collected hourly water samples over a 24-h time series from the tidal reach of the Pearl River Estuary (PRE) and explored the dynamics of both prokaryotic and eukaryotic microbial diversity and their co-occurrence networks during a complete tidal cycle by utilizing RNA-derived cDNA combined with third-generation (PacBio) amplicon sequencing. This approach targets the active microbial community (by using RNA) while providing high taxonomic resolution (by full-length sequencing of 16S rRNA gene / ITS regions). Our results revealed that salinity was the dominant driver of the changes of microbial diversity and community compositions in the tidal reach, with a significant decrease in microbial alpha diversity as salinity increased. Furthermore, we observed a non-linear relationship between salinity and microbial network complexity and stability. Notably, network stability in both single- and cross-domain networks was positively correlated with connectance and P/N (the positive-to-negative edge ratio). Additionally, cross-domain networks exhibited higher complexity (node numbers, total links, and average node degree), but lower network stability (robustness and vulnerability) compared to single-domain co-occurrence networks. Overall, our findings provide comprehensive insights into how periodic salinity fluctuations regulate microbial community structure and cross-domain interactions in dynamic estuarine environments.

**Supplementary Information:**

The online version contains supplementary material available at https://doi.org/10.1007/s00248-026-02804-4.

## Introduction

Microbes play a crucial role in estuarine ecosystems by driving biogeochemical cycling and serving as essential contributors to ecosystem functioning [1–3]. The tidal reaches, located in the estuarine front, are characterized by dynamic salinity fluctuations due to periodic tides [4]. However, how these periodic salinity fluctuations in the tidal reaches affect microbial diversity, composition, and ecological network structure remains poorly understood. This knowledge gap limits our thorough comprehension of how periodic tidal cycles influence microbial communities in estuarine ecosystems.

Salinity has been identified as a pivotal environmental factor influencing the diversity and composition of microbial communities in aquatic environments [5]. The impacts of elevated salinity on microbial diversity are diverse, encompassing reductions in diversity [6, 7], no observable effects [8], or even a peak at moderate salinity levels [9–11]. Moreover, salinity shapes community structure by selectively enriching salt-tolerant taxa while excluding those with low salinity tolerance [12]. Previous studies have shown that *Alphaproteobacteria*, *Gammaproteobacteria*, *Dinoflagellates*, and *Haptophyta* are generally well-adapted to high salinity conditions in aquatic environments [13–15]. Notably, the SAR11 clade (*Alphaproteobacteria*) comprises multiple ecotypes with distinct salinity preferences. For instance, subclade Ic exhibits exhed genomic adjustments to deep-sea environments [ibit^16^]; subclade IIIb (LD12) has lost osmoregulation genes, explaining its restriction to freshwater [17, 18]; and subclade IIIa.3, which inhabits brackish zones, possesses genes for solute transport and iron utilization [19]. Although numerous studies have highlighted the role of salinity in shaping microbial diversity and composition [20–22], we still know little on how periodic salinity fluctuations affects the network complexity and stability of microbial communities in the tidal reach.

Microorganisms, including both prokaryotes and microeukaryotes, do not exist in isolation in the ecosystems, instead, they engage in intricate interactions with each other [23]. However, it is challenging to directly monitor or study ecological interactions between microorganisms. In recent years, the inference of microbial potential interactions using statistical methods based on the co-abundance of microbes has been widely performed and demonstrated to be an effective tool for examining the associations and organization of microbial communities [24–26]. Recent studies have highlighted the significant impact of salinity on microbial ecological networks across various habitats [7, 11, 27]. For example, lower shifts in salinity could change the network stability of microeukaryotic communities in freshwater [7]. In Tibetan Plateau lakes, salinity has been shown to decrease bacterial diversity but increase the network complexity of bacterial communities [11]. Soil salinization has been found to enhance the network stability of fungal communities rather than bacterial communities in the Taklamakan Desert [28]. While most previous studies have predominantly focused on single-domain networks, examining specific types of microbes such as bacteria, archaea, fungi, or protists [27–32], is crucial to recognize that intricate interactions occur among microorganisms spanning diverse trophic levels. Exploring cross-domain networks can enrich our comprehension of microbial communities by offering a more holistic view.

DNA-based amplicon sequencing is a powerful tool for identifying a wide array of microorganisms in the environments [33]. However, environmental relic DNA (DNA from dead cells) can persist in the environments and impact DNA-based amplicon sequencing, leading to an inaccurate depiction of microbial diversity and composition [34]. In contrast, RNA is known to be stable in active cells but will be rapidly degraded after cell lysis [35]. Consequently, RNA-based (cDNA) amplicon sequencing exclusively captures transcripts from only active cells, thereby mitigating the amplification of environmental relic DNA [36, 37]. Additionally, compared with short-read sequencing, the third-generation sequencing can cover the full-length of 16S rRNA gene and internal transcribed spacer (ITS) region (the most widely used molecular maker for profiling prokaryotic and eukaryotic microbes, respectively). Unlike partial gene sequencing, the full-length 16S rRNA gene/ITS sequencing can provide higher taxonomic resolution [38, 39], thus may provide more detailed insights into the microbial communities.

In this study, we collected 24 time-series water samples at hourly intervals from the tidal reach of the Pearl River Estuary (PRE), which serves as a crucial transition zone linking terrestrial and riverine inputs to the South China Sea [40]. Utilizing the third-generation RNA amplicon sequencing technique, we aim to explore the following questions: (i) How does periodic salinity fluctuation of tidal reach affect the diversity and community composition of prokaryotic and eukaryotic microorganisms? (ii) How do the complexity and stability of single-domain and cross-domain networks respond to periodic salinity fluctuation? These explorations would enhance our understanding of microbial responses to environmental fluctuations, thereby contributing to the elucidation of mechanisms that drive dynamics of microbial communities in the tidal reaches.

## Materials and Methods

### Sample Collection and Physicochemical Measurement

The Pearl River Estuary (PRE) is a key subtropical estuary where the Pearl River meets the South China Sea, forming a pronounced and dynamic salinity gradient. The mixing of freshwater and seawater supplies both terrestrial and marine-derived organic matter, creating a nutrient-rich habitat that sustains diverse and active microbial communities. These distinct environmental gradients make the PRE an ideal natural system for studying how salinity fluctuations shapes microbial community structure and ecological interactions.

Samples were collected from Yamen Outlet (22°18' N, 113° 04' E, located at Huangmao Sea, Guangdong Province, China, in March 2022 (Fig. S1). The sampling sites represents the upper, river-dominated section of the Pearl River Estuary. The sampling area exhibits a convergent geometry, narrowing sharply from 35 km at the mouth to only 1.9 km at the apex—the confluence of the Yamen Outlet [41]. This geographical setting places our sampling area close to the freshwater source, resulting in consistently low salinity (0–6 PSU, Fig.S2). Over the course of a complete tidal cycle, we collected 24 hourly bottom water samples from depths ranging between 9 and 11 m. Each water sample (~ 10 L) was filtered through a 3-μm and 0.22-μm membrane filter (Pall Life Sciences, Ann Arbor, MI, USA) via peristaltic pump to collect microbes. Subsequently, each membrane filter was transferred to the sterile bag and promptly stored at −80℃ until RNA extraction. The filtered water was aliquoted into the sterile 50 mL tubes and stored at 4℃ for subsequent physicochemical analysis.

RNA was extracted from both the 0.22-μm and 3-μm filters for all 24 time points. Preliminary analysis revealed that the free-living (0.22-μm) and particle-associated (3-μm) communities did not differ significantly in alpha diversity (*P* > 0.05, PERMANOVA). Furthermore, distance-based redundancy analysis (db-RDA) indicated that both community types were primarily and similarly structured by salinity (Fig. S3). Therefore, sequence data from both 0.22-μm and 3-μm fractions at each time point were combined for all downstream analyses.

Physicochemical properties, including temperature, depth, salinity, turbidity, pH, Oxidation–Reduction Potential (ORP) were measured in situ with a Conductivity Temperature Depth Rosette system (CTD). Total organ carbon (TOC), dissolved nitrogen (DN), ammonia nitrogen (NH_4_^+^), dissolved Phosphorus (DP), phosphate (PO_4_^3−^), and cell number were measured according to standard protocols as previously described [42, 43].

### RNA Extraction and Sequencing

Total RNA was extracted from the filters according to the standard kit protocol (RNeasy PowerSoil Total RNA Kit, QIAGEN, Germany). The quality and concentration were measured with a NanoDrop 2000 UV–Vis spectrophotometer (Thermo Fisher, USA). Furthermore, the RNA samples were submitted to Magigene Company (Guangdong, China) for full-length sequencing of the 16S rRNA gene and ITS region using the PacBio Sequel II platform.

The full-length 16S rRNA gene was amplified with the primer pairs 27 F (AGRGTTYGATYMTGGCTCAG) and 1492R (RGYTACCTTGTTACGACTT) [44]. Additionally, the amplification of the full-length ITS region was carried out with the primer pairs ITS9munngs (TACACACCGCCCGTCG) and ITS4ngsUni (CCTSCSCTTANTDATATGC) [45]. After the amplification, the resulting amplicon products were purified, barcoded, and sequenced on PacBio Sequel II platform as described previously [46]. The raw sequences were deposited in the National Center for Biotechnology Information (https://www.ncbi.nlm.nih.gov/) under the accession number PRJNA1310827 (16S rRNA genes) and PRJNA1310809 (ITS region).

### Amplicon Analysis

Amplicon sequencing data obtained from 24 samples were analyzed using the USEARCH pipeline (version 11; http://www.drive5.com/usearch/) through the following pipeline: (1) Low-quality reads and adapters were first removed from the raw data using CUTADAPT v2.4 software [47]. (2) Sequences that were less than 500 bp or with expect error > 1 were discarded before further analysis. (3) After dereplication, prokaryotic 16S rRNA gene sequences were clustered into amplicon sequence variants (ASVs) at 100% similarity threshold by UNOISE3 algorithm [48], whereas eukaryotic ITS sequences were clustered into operational taxonomic units (OTUs) at 97% similarity threshold [49]. (4) The prokaryotic ASVs and eukaryotic OTUs were taxonomically annotated with the Ribosomal Database Project (RDP) Classifier in QIIME 2 platform [50] against SILVA 138 database [51] and UNITE 8.0 database [52], respectively. (5) To normalize the sampling effort, ASV and OTU tables were rarefied to 4,698 sequences for prokaryotic communities and 11,992 sequences for eukaryotic communities according to the minimum sequence number of 24 water samples with *rrarefy* function in the *vegan* package [53].

The alpha diversity was calculated as species richness (Hill number q = 0), exponential of the Shannon entropy (Hill number q = 1), and inverse Simpson index (Hill number q = 2) [54, 55]. Scatter plots were used to investigate the relationship between alpha diversity and salinity. Given the nonlinear nature of this relationship, we employed generalized additive models (GAMs) to analyze the relationship between alpha diversity and salinity via the *mgcv* package [56]. Correlations between alpha diversity and salinity were quantified by Spearman correlation coefficient. To elucidate the temporal variation of alpha diversity, we initially developed a linear regression model with salinity, temperature, depth, turbidity, cell number, TOC, DN, DP, NH_4_^+^, and PO_4_^3−^ as independent variables. The best regression model was selected using the Akaike information criterion (AIC) through the step function in the *stats* package. Subsequently, the lmg metric in the *relaimpo* package [57] was employed to assess the relative contributions of the selected environmental factors.

In order to examine the variation in microbial community composition in 24 samples, we plotted the relative abundance of the top 10 phyla and clustered the samples using the 'UPGMA' method based on their Bray–Curtis distance via *ggtree* package [58] at the ASVs/OTUs level. We found that the 24 samples formed three distinct clusters (Fig. S4), which were consistent with observed variations in environmental salinity. As a result, we further performed subsequent analysis by categorizing the 24 samples into three groups based on salinity levels, specifically low salinity (0.20–1.03 PSU), medium salinity (1.81–4.71 PSU), and high salinity (4.72–6.68 PSU).

Prokaryotic and eukaryotic community compositions were visualized via the Principal Coordinates Analysis (PCoA) based on the Bray–Curtis distance using the *vegan* and *ggplot2* packages [59]. The distance-based redundancy analysis (db-RDA) was implemented to investigate the effect of abiotic (environmental) and biotic variables on prokaryotic and eukaryotic communities. Before RDA analysis, species data were Hellinger-transformed, and environmental variables were standardized using z-score normalization to eliminate scale effects. We selected statistically important environmental variables by forward selection (*P* < 0.05) using ordiR2step function in *vegan* package. The hierarchical partitioning analysis was performed to quantitatively evaluate the relative importance of the environmental factors via the *rdacca.hp* package [60, 61]. The Mantel test between environmental variables, microbial diversity and microbial community were performed in *linkET* package [62].

To enhance the robustness of the construction of co-occurrence networks, rare ASVs/OTUs occurring in less than eight samples were discarded. After filtering, 1,706 prokaryotic ASVs and 673 eukaryotic OTUs together with the 12 environmental variables were used to construct cross-domain network using the extended local similarity analysis (eLSA) [63, 64]. In addition, single-domain networks (prokaryote-only, eukaryote-only) were separately generated via eLSA [63, 64]. To investigate how the periodic salinity fluctuation affects the correlations between different domains, we only kept the cross-domain edges in the cross-domain networks. We applied iDIRECT to distinguish direct from indirect relationships in cross-domain and single-domain networks [65]. Furthermore, sub-network for each sample was generated using the subgraph function of the *igraph* package [66]. We retained only strong correlation edges (absolute local similarity score |LS|> 0.8, *P*-value < 0.001, Q < 0.001) for downstream analysis.

Network topological properties, including node number (*n*), edge number (*L*), average degree (average *K*), average path length (GD), graph diameter, connectance, average clustering coefficient (average *CC*), betweenness centralization, degree centralization and modularity were calculated with the *igraph* package [66, 67]. Average degree is defined as the average number of edges per node in the network. Higher average degree means a more complex network [67]. Nodes were classified into four topological roles based on their within-module degree (Z_i_) and among-module connectivity (P_i_): module hubs (Z_i_ > 2.5, P_i_ < 0.62), connectors (Z_i_ < 2.5, P_i_ > 0.62), network hubs (Z_i_ > 2.5, P_i_ > 0.62) and peripherals (Z_i_ < 2.5, P_i_ < 0.62) [68]. Nodes apart from peripherals in each co-occurrence network were defined as potential keystone species [69]. To understand how salinity affected the stability of the co-occurrence network, two network indices (robustness and vulnerability) were used to predict the stability of cross-domain network and single-domain network. The robustness of a network is defined as its resistance to node loss after random or target node removal. Robustness quantifies the resistance of ecological network to species extinction and was measured as the proportion of the remaining taxa in the network after randomly removing 50% nodes [27]. The vulnerability of a network is indicated by the maximal vulnerability observed among its individual node, which measures their relative contribution to the global efficiency of the network [27]. We conducted generalized additive models (GAMs) to investigate the relationship between network properties and salinity via the *mgcv* package [56]. To further determine whether topological properties influence the stability of co-occurrence networks, Spearman’s rank correlation coefficients were calculated between network stability metrics and topological features. Correlation matrices were visualized using heatmaps implemented in the *corrplot* package[70]. All statistical analyses and data visualizations (except where otherwise specified) were implemented using *ggplot2* within R (version 4.2.2) [59].

## Results

### Microbial Diversity and Community Composition

After data filtering and screening, 462,919 full-length 16S rRNA gene amplicon reads (prokaryotes) and 857,398 full-length ITS region amplicon reads (eukaryotes) were obtained from 24 water samples collected in the tidal reach of the Pearl River Estuary. After clustering sequences at a 100% similarity level, we obtained 6,569 prokaryote unique amplicon sequence variants (ASVs), while 97% similarity clustering identified 7,079 eukaryotic operational taxonomic units (OTUs).

**Fig. 1:**
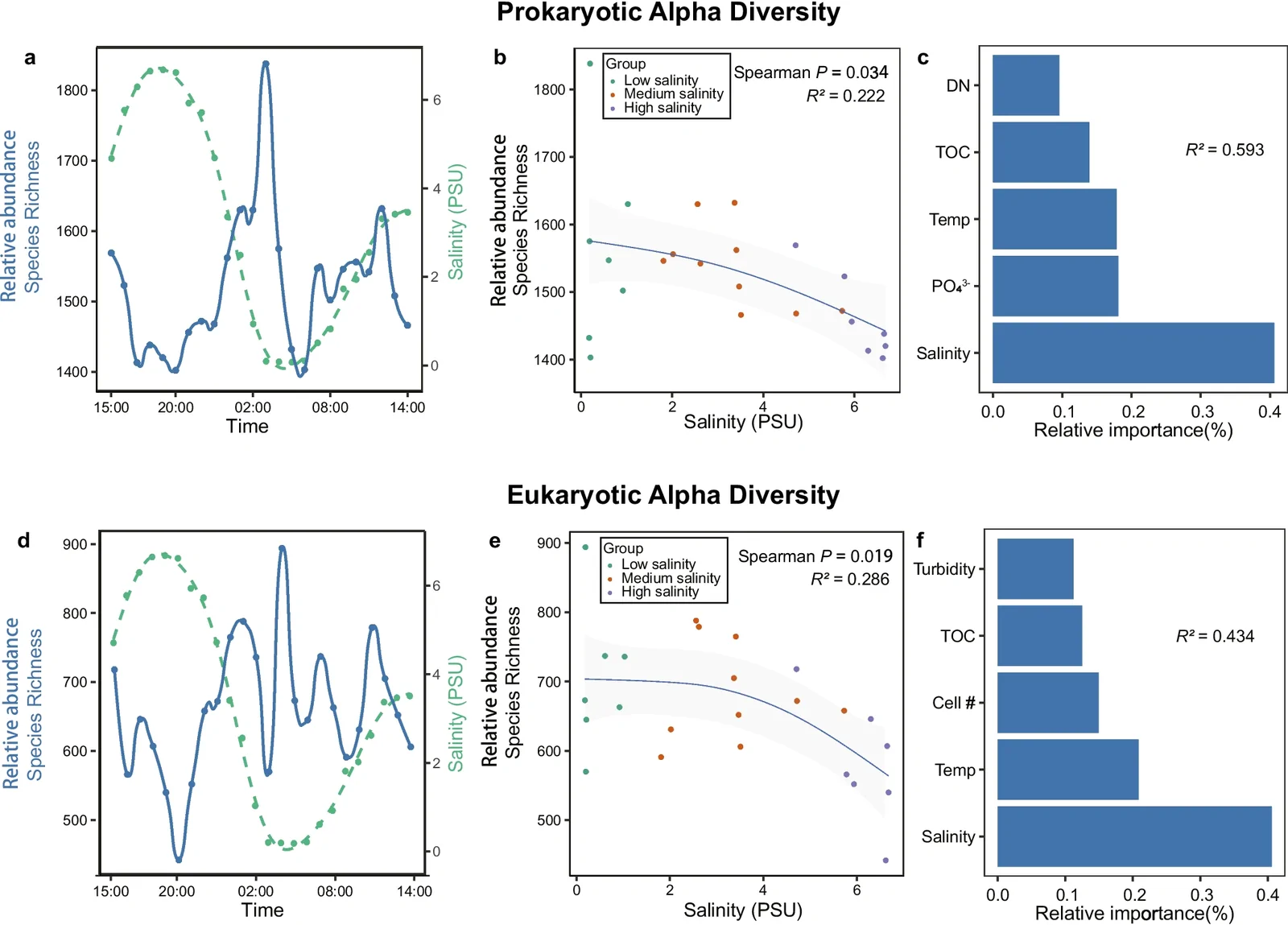
Variation of species richness and salinity of 24 samples over time in prokaryotic **(a)** and eukaryotic **(d)** communities. Relationships between salinity and species richness of prokaryotic **(b)** and eukaryotic communities. The relative contributions of different environmental factors to microbial alpha diversity **(c, f)**.* R*^*2*^ represents the total variance that could be explained by selected factors. Temp, temperature

Species richness decreased over time as salinity increased (Figs. 1a, d). To investigate the effect of salinity on alpha diversity, we conducted generalized additive models (GAMs) to investigate the relationship between species richness and salinity (Fig. S5). Salinity exhibited significant negative correlation with species richness in both prokaryotic (*R*^*2*^ = 0.222, *P* < 0.05) and eukaryotic community (*R*^*2*^ = 0.286, *P* < 0.05) (Figs. 1b, e). Most of the variation in prokaryotic (59.3%) and eukaryotic community alpha diversity (43.4%) could be explained by environmental factors (Fig. 1c, f), and salinity was the most important factor contributing 40.6% and 40.4% to the total explained variation, respectively (Fig. 1c, f).

**Fig. 2:**
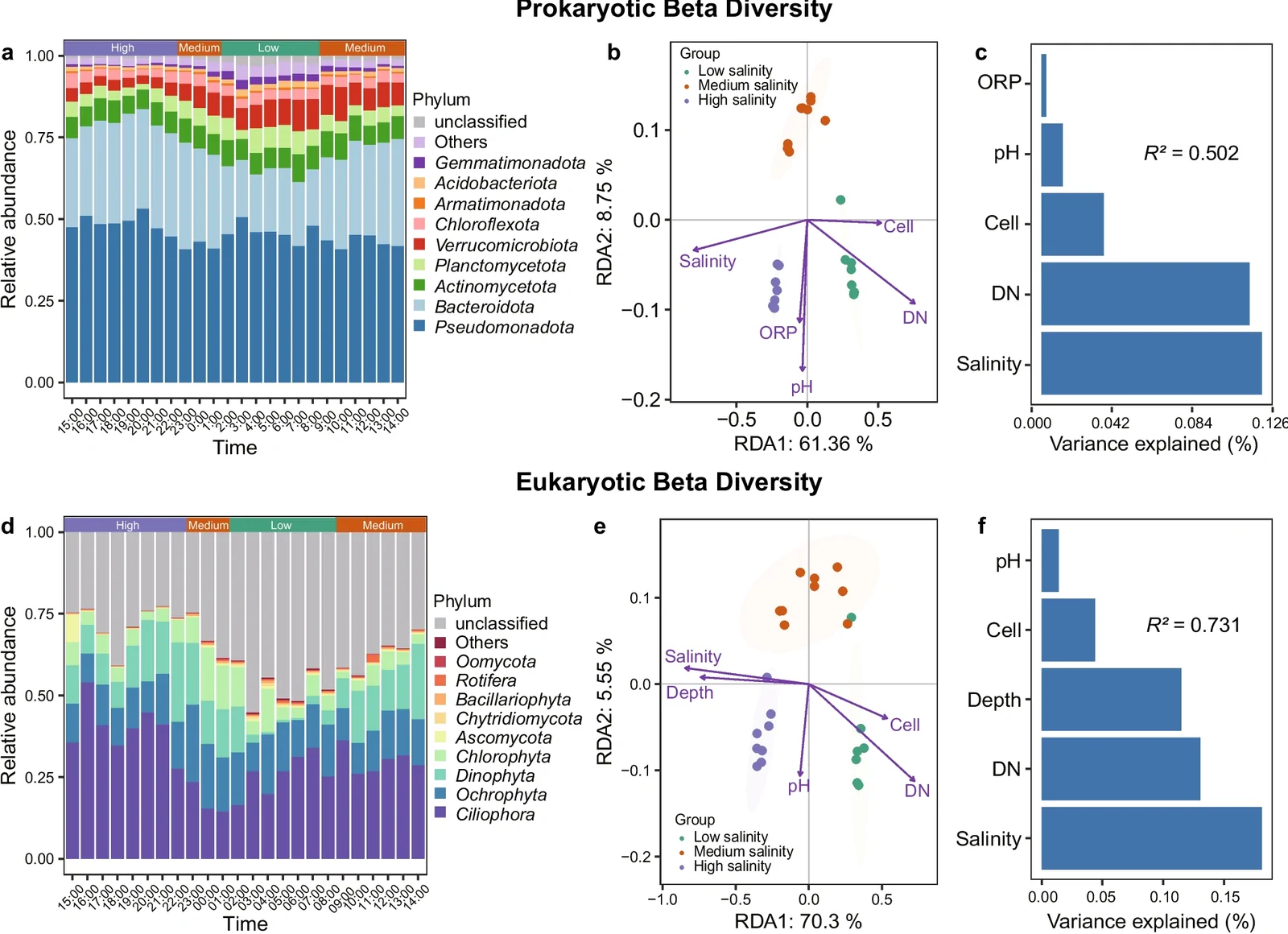
The relative abundances of different phyla in the prokaryotic **(a)** and eukaryotic **(d)** community composition among 24 samples. Top 10 abundant phyla were showed and the rest were incorporated and exhibited as “Others”. The distance-based redundancy analysis (db-RDA) results of prokaryotic **(b)** and eukaryotic **(e)** community. The relative variance explained by each axis was labelled in the axis title. Bar plot were shown below db-RDA presenting the variation explained by each environmental factor in prokaryotic **(c)** and eukaryotic **(f)** community. High, high salinity; Medium, medium salinity; Low, low salinity

Microbial community compositions in each sample were depicted in Fig. 2. We found that the prokaryotic community was dominated by *Pseudomonadota*, with 46.09% relative abundance on average, followed by *Bacteroidota* (26.65%), *Actinomycetota* (6.74%), and *Verrucomicrobiota* (5.91%) (Fig. 2a). The eukaryotic community was largely occupied by four major phyla, including *Ciliophora* (30.56%), *Ochrophyta* (13.76%), *Dinophyta* (11.26%) and *Chlorophyta* (6.18%), accounting to 61.77% of the total relative abundance (Fig. 2d). The community structure of prokaryote and eukaryote at the phylum level varied among different salinity. In response to increasing salinity, there was a significant decrease (Bayesian, *P* < 0.05,) in the relative abundances of *Verrucomicrobiota, Planctomycetota*, *Gemmatimonadota*, *Acidobacteriota*, and *Chytridiomycota*. Conversely, the relative abundances of *Ciliophora*, *Dinophyta* and *Bacteroidota* showed a gradual increase (Bayesian, *P* < 0.05) (Fig. S6a, b). To further explore taxonomic composition below the phylum level, we conducted order-level analyses for both prokaryotes and eukaryotes (Fig. S7). Within *Ciliophora*, the three most abundant orders—*Tintinnida*, *Cyclotrichida*, and *Choreotrichida*—all increased with salinity, consistent with phylum-level trends (Fig. S7). Within *Pseudomonadota*, *Burkholderiales* (*Betaproteobacteria*) decreased with salinity, while *Rhodobacterales* (*Alphaproteobacteria*) increased (Fig. S7).

During a complete tidal cycle, the environmental factors exhibited significant fluctuations over time (Fig. S2). During ebb tide (falling sea level), the salinity level decreased, and the lowest values coincided with a high increase in turbidity, DN, and to a lesser extent, phosphate. Whereas flood tide (rising sea level) featured increasing salinity with lower turbidity and nutrient levels.

Distance-based redundancy analysis (db-RDA) was performed to evaluate the effects of biotic and abiotic environmental variables on prokaryotic and eukaryotic community structures (Fig. 2b, e). The db-RDA analysis indicated that both prokaryotic and eukaryotic communities were primarily influenced by salinity and dissolved nitrogen (DN) (*P* < 0.05; Fig. 2b, e). Furthermore, variance partitioning analysis confirmed that salinity was the most important drivers of both prokaryotic and eukaryotic communities (Fig. 2c, f). Mantel analyses further revealed significant associations between both alpha diversity and community dissimilarity with multiple environmental variables, salinity being the most prominent (Fig. S8). Accordingly, the PCoA (Principal Coordinates Analysis) result confirmed that the strong effect of salinity on the microbial community, which both prokaryotic and eukaryotic community compositions were clustered according to salinity (Fig. S9).

In estuarine systems, pH reflects metabolic and carbonate chemistry, while DN indicates terrestrial nutrient input. Although both variables can influence microbial communities, their apparent importance in the db-RDA likely arises from co-variation with salinity driven by tidal forcing. However, across all analyses (alpha diversity, community composition, network stability), salinity consistently emerged as the strongest and most consistent predictor.

### Changes in the Complexity of Microbial Communities Along Salinity Gradient

In order to characterize and evaluate the co-occurrence interactions of microbial community across a salinity gradient, we constructed three co-occurrence networks: one for the prokaryotic community, one for the eukaryotic community, and one that integrated both prokaryotic and eukaryotic communities (cross-domain). There were 827 nodes linked by 3,559 edges and 378 nodes linked by 2,409 edges for single-domain networks of prokaryote and eukaryote, respectively. The cross-domain network integrated 1,123 nodes (692 prokaryotic and 431 eukaryotic) connected by 8,701 edges, 46.3% of which represented positive associations (Table 1). To study the relationships between salinity and network topological parameters, we generated sub-networks for each water sample by keeping ASVs/OTUs associated with specific samples and all edges among them in the prokaryotic, eukaryotic, and cross-domain co-occurrence networks.

**Table 1:** Topological characteristics of single-domain and cross-domain networks

Network indexes	Prokaryote	Eukaryote	Cross-domain
*n*	827	378	1123
*L*	3559	2409	8701
Average *K*	8.607	12.746	15.496
GD	3.288	3.051	1.733
Graph diameter	8.995	12.744	10.012
Connectance	0.010	0.033	0.014
Average CC	0.071	0.070	0.018
Betweenness centralization	0.078	0.054	0.073
Degree centralization	0.092	0.136	0.119
Modularity	0.356	0.287	0.426

**Fig. 3:**
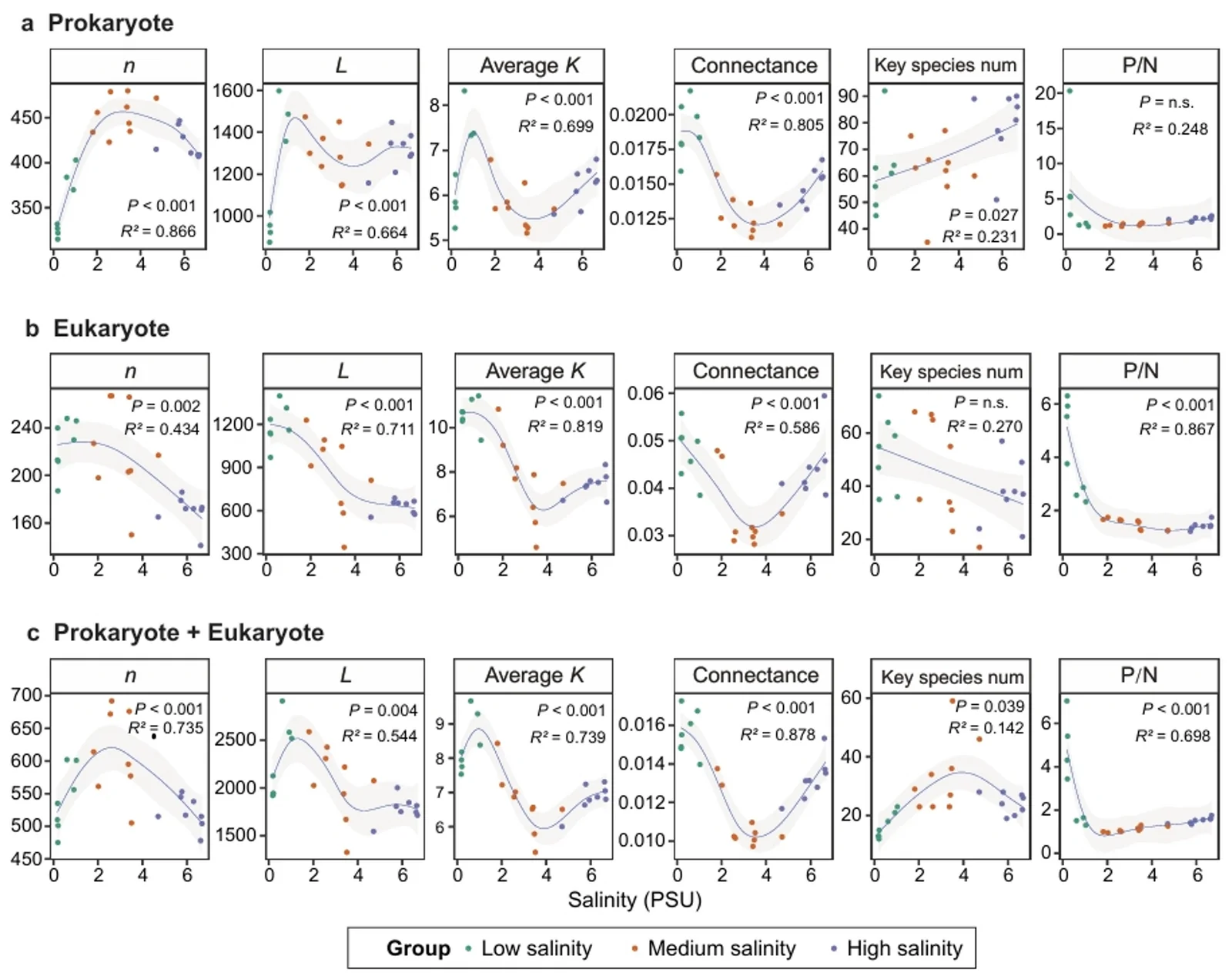
Relationship of network complexity with salinity and multiple network topological parameters. (**a**) prokaryote, (**b**) eukaryote, (**c**) cross-domain (prokaryote + eukaryote). Network topological parameters include node numbers (n), total links (L), average node degree (average K), connectance, key species number (Key species num), and P/N (ratio of the positive-to-negative edge ratio). The adjusted R2 and P values are shown.

To determine how salinity affected microbial network complexity and stability, various network topological parameters of subnetworks were regressed against salinity. We found that prokaryotic, eukaryotic, and cross-domain networks had different properties and responded differently to salinity. The relationships between the variables *n*, *L*, and average *K* of the prokaryotic and cross-domain sub-networks displayed a non-linear pattern with salinity, which showed an initial increase, followed by a decrease, and then a subsequent increase with increasing salinity (Fig. 3a, c). For eukaryotic network, we found that network size (total number of nodes, *n*) and total links (total number of links, *L*) significantly decreased as salinity increased, indicating that the eukaryotic sub-network at higher salinity included fewer nodes and fewer interactions between nodes (Fig. 3b). Compared with the single-domain sub-networks, we found cross-domain sub-networks obtained higher *n*, *L*, and average *K*, suggesting that there were more microorganism associations in cross-domain sub-networks. That is, cross-domain sub-networks had higher complexity. The key species number of prokaryotic and cross-domain sub-networks increased with increasing salinity (prokaryote: *R*^*2*^ = 0.231, *P* = 0.027; cross-domain: *R*^*2*^ = 0.142, *P* = 0.039) (Fig. 3a, c). Nevertheless, the key species number of eukaryotic community showed no significant correlations with salinity (*R*^*2*^ = 0.270, *P* = 0.098) (Fig. 3b). The identified keystone taxa predominantly belonged to the eukaryotic phyla *Ciliophora*, *Ochrophyta*, *Chlorophyta* and *Dinophyta* and prokaryotic phyla *Pseudomonadota*, *Bacteroidota*, *Verrucomicrobiota* and *Actinomycetota* (Fig. S10). The ratio of P/N (the positive-to-negative edge ratio) exhibited a consistent trend in prokaryotic, eukaryotic, cross-domain networks, declining as salinity increased (Fig. 3).

### Changes in the Stability of Microbial Communities Along Salinity Gradient

**Fig. 4:**
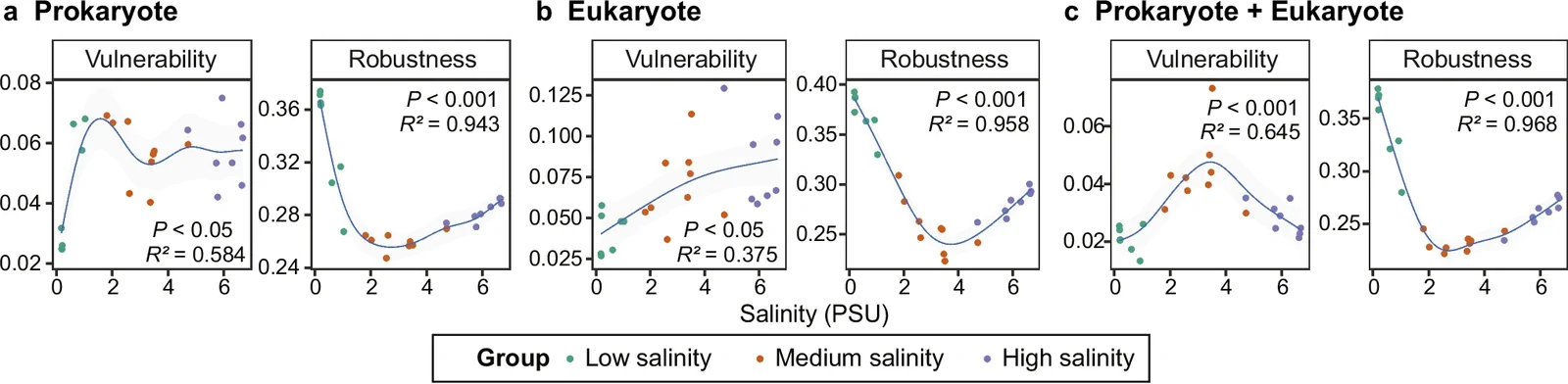
Relationship of network stability with salinity and multiple network topological parameters. **(a)** prokaryote, **(b)** eukaryote, **(c)** cross-domain (prokaryote + eukaryote). Network topological parameters include vulnerability and robustness. Network robustness was calculated as the remained proportion of taxa after randomly removing 50% of the taxa from each sub-network. Network vulnerability was calculated as the maximum node vulnerability in each sub-network. The adjusted *R*^*2*^ and *P* values are shown.

We characterized the stability of microbial sub-networks in response to salinity using the metrics of vulnerability and robustness. The stability of prokaryotic, eukaryotic, and cross-domain sub-networks showed a consistent non-linear change trend with increasing salinity (Fig. 4). Compared with single-domain co-occurrence networks, cross-domain network displayed numerically slightly lower robustness and higher vulnerability, indicating lower network stability in the tidal reach under periodic salinity fluctuations (Fig. 4). Both prokaryotic and cross-domain co-occurrence networks exhibited higher node numbers (*n*) and total links (*L*), but lower robustness and higher vulnerability, indicating a negative correlation between network complexity and network stability (Figs. 3, 4).

**Fig. 5:**
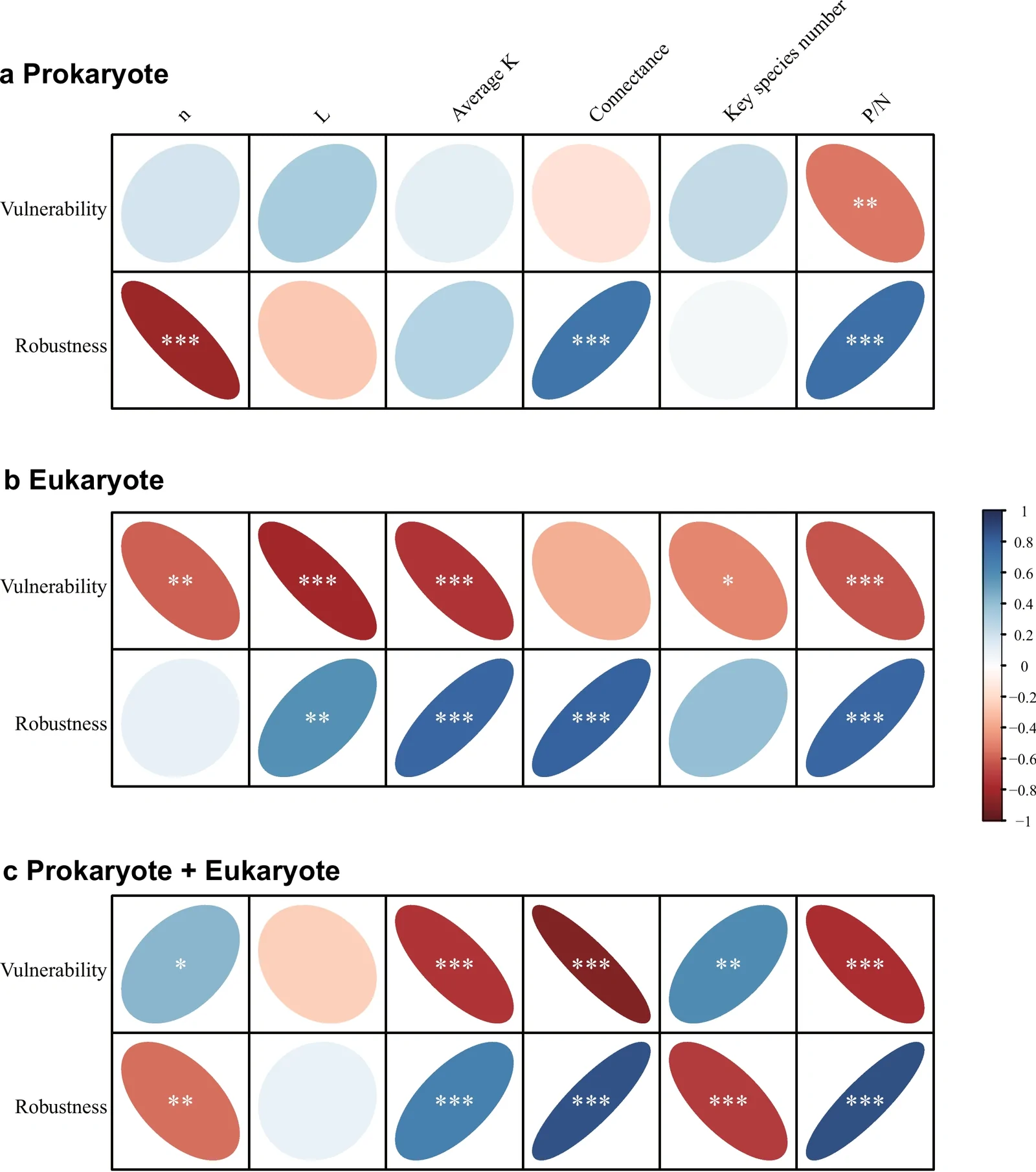
Spearman correlation (*, *P* < 0.05; **, *P* < 0.01; ***, *P* < 0.001) of network robustness and vulnerability with different network properties, including *n*, *L*, average *K*, connectance, modularity, key species number, and P/N (the ratio of positive/negative edges)

To further investigate whether network parameters can impact network stability, we calculated the correlation between network parameters and network stability. In the prokaryotic networks, connectance exhibited a significant positive correlation with P/N, whereas *n* was negatively associated with robustness (Fig. 5a). Furthermore, we observed no significant relationship with vulnerability in the prokaryotic network (Fig. 5a). Network stability showed significant positive relationships with network complexity, where the stability increased with *L*, Average *K*, connectance, and P/N in eukaryotic network (Fig. 5b). We found that network stability showed significant relationships with network complexity except *L* (total links) in cross-domain networks (Fig. 5c), where network stability increased with average *K*, connectance, and P/N, but decreased with *n* and key species number in cross-domain network.

## Discussion

Our hourly-resolution approach reveals that substantial shifts in microbial diversity and composition can occur within a single tidal cycle in response to rapid salinity fluctuations. Notably, although salinity varied within a relatively narrow range (0–6 PSU), we observed pronounced shifts in microbial diversity, community composition, and network properties across this gradient. Most previous studies on estuarine microbial communities have focused on seasonal or monthly scales [71–73] whereas our hourly-resolution approach reveals that substantial shifts in microbial diversity and composition can occur within a single tidal cycle in response to rapid salinity fluctuations.

### Salinity Shapes the Diversity and Community Composition of Prokaryotes and Eukaryotes

Salinity has been shown to be a key driver of microbial diversity [7, 11], which is consistent with our findings that salinity significantly affected microbial alpha and beta diversity (Figs. 1, 2). For example, Mohamed et al. (2010) demonstrated that the composition of fungal communities in estuarine sediments shifts substantially along salinity gradients, with distinct assemblages in freshwater, brackish, and marine zones [74]. In the Pearl River Estuary specifically, Wang et al. (2019) showed that salinity was one of the main drivers of both alpha and beta diversity of fungi [75]. Notably, the responses were domain-specific: eukaryotic richness declined at a more gradual rate than prokaryotic richness (Fig. 1b, e). This divergence may be attributed to the broader physiological plasticity and structural adaptations (e.g., robust cell walls in fungi) found in many eukaryotic lineages, which confer greater resilience to osmotic stress [76, 77].

In estuarine ecosystems, multiple environmental factors co-vary with tidal forcing. For instance, prokaryotic alpha diversity increased with turbidity and cell concentration, whereas eukaryotic alpha diversity showed a unimodal response (Fig. S11). Dissolved nitrogen (DN) exhibited correlation patterns with network properties that were qualitatively similar to those of salinity (Fig. S12, Fig. S13), and pH also appeared important in db-RDA biplots. However, these patterns likely reflect co-variation driven by the same tidal hydrological process rather than independent effects. Variance partitioning confirmed that salinity had the highest unique contribution to both alpha diversity (Figs. 1c, f) and community composition (Figs. 2c, f), and it consistently emerged as the strongest predictor across all analyses (alpha diversity, community composition, and network stability). Therefore, while other variables play secondary roles, salinity remains the most direct and ecologically interpretable indicator of tidal forcing.

Although the physiological mechanisms underlying differential salinity tolerance in fungi remain incompletely understood, several adaptive strategies have been proposed: First, many fungi can balance internal water potential by actively taking in ions such as potassium and storing them in vacuoles, thereby preventing cellular dehydration under saline conditions [78, 79]. Second, yeasts and filamentous fungi can produce or absorb small organic molecules called osmolytes—glycerol is a common example [78]. These compounds protect proteins and enzymes from salt damage without interfering with normal cell functions [80].Third, filamentous taxa may undergo mechanical reinforcement of the cell wall, including increased wall thickness under saline conditions [81].

Despite the overall decline in diversity, certain lineages exhibited opposing trends: *Pseudomonadota*, *Bacteroidota*, *Dinophyta*, and *Ciliophora* increased in relative abundance with salinity (Fig. 2). These observations suggest that the effect of salinity extends beyond general suppression to differentially influence phylogenetic groups, thereby driving shifts in community composition. Within the prokaryotic community, *Pseudomonadota*, *Bacteroidota*, and *Actinobacteriota* collectively accounted for 69.8–87.8% of the total relative abundance across all samples. The relative abundances of *Pseudomonadota* and *Bacteroidota* were positively correlated with salinity (Fig. 2), consistent with their known physiological adaptations. For instance, the "salt-in" strategy—regulating K⁺ uptake and Na⁺ efflux to balance intracellular osmolarity—enables many members of these phyla to tolerate salinity fluctuations [82]. In our study, the relative abundances of *Pseudomonadota* increased significantly along the salinity gradient (*P* < 0.05, Fig. S6). Multiple studies across distinct coastal habitats have reported positive correlations between salinity and the relative abundances of *Alphaproteobacteria*, *Deltaproteobacteria*, and *Gammaproteobacteria* (three classes within the phylum *Pseudomonadota*) [83–85]. Conversely, species belonging to *Acidobacteria* had preferences for low salinity, which were also reported in other studies where they were largely replaced by *Pseudomonadota*, *Bacteroidota* and *Bacillota* with increasing stresses (e.g., osmotic pressure or organic resources) [86].

In this study, domain phyla in eukaryotic community were in accordance with previous researches in PRE [85, 87, 88], mainly dominated by *Ciliophora*, *Ochrophyta*, *Dinophyta* and *Chlorophyta* (Fig. 2d). *Dinophyta* was positively correlated with salinity (Fig. S6), which was consistent with a former study in tropical coastal lagoon [89]. Within aquatic environments, phytoplankton (for example, *Ochrophyta*, *Dinophyta*, and *Chlorophyta*) are the dominant primary producers and responsible for almost 50% of global carbon fixation [90, 91]. The fact that *Dinophyta* and *Bacteroidota* exhibited similar distribution patterns along the salinity gradient (Fig. S6) may be related to the dependence of the latter group on organic matter produced by this phytoplankton group. Furthermore, *Bacteroidota* has been widely reported as a typical component of the dinoflagellate phycosphere [92]. *Ciliophora* were strongly affected by salinity, and their abundance increased with increasing salinity. These findings aligned with previous research, which had also indicated that the abundance of *Ciliophora* occurred a peak at mesohaline and polyhaline regions [93, 94]. The inflow of seawater into PRE resulted in an increase in salinity. Simultaneously, this influx brought a significant amount of nutrients including calcium, nitrate, phosphate and so on, which were essential for phytoplankton growth which provided food for *Ciliophora* resulting in the high abundance in higher salinity (Fig. 2d) [95]. We posit that salinity acted as a critical environmental filter in structuring the ciliate community. Li et al. (2019) found that certain ciliate groups, such as the order *Tintinnida*, possess broad salinity tolerance (e.g., found across 0.7–34.0 PSU in the Pearl River Estuary) [96]. Under high-salinity conditions, such tolerant taxa were positively selected and became dominant. Conversely, many freshwater or euryhaline taxa that cannot tolerate high salinity were suppressed. This community assembly process, where tolerant taxa thrive, and sensitive ones are filtered out, likely explains our observed pattern: a positive correlation between the overall relative abundance of the phylum *Ciliophora* and increasing salinity. To further explore the taxonomic composition, we conducted an order-level analysis of the eukaryotic community (Fig. S7). The three most abundant ciliate orders identified were *Tintinnida*, *Cyclotrichida*, and *Choreotrichida*. Notably, the relative abundances of all these orders showed a clear positive correlation with increasing salinity, mirroring the trend observed at the phylum level (Fig. 2, Fig. S7).

Two orders within *Pseudomonadota*—*Burkholderiales* (*Betaproteobacteria*) and *Rhodobacterales* (*Alphaproteobacteria*)—showed opposite trends along the salinity gradient (Fig. S7). The relative abundance of *Burkholderiales* decreased with increasing salinity, while *Rhodobacterales* increased (Fig. S7)*.* This differential response pattern is consistent with previous findings reported by Li et al. (2023) [6], who demonstrated that *Alphaproteobacteria* become increasingly dominant under elevated salinity stress while *Betaproteobacteria* gradually lose their dominance. We found that the relative abundance of SAR11 increased with salinity (Fig. S7), a pattern consistent with observations in the Vistula Lagoon (an inner estuary of the Baltic Proper), where SAR11 clade I/II abundance was strongly positively correlated with salinity across a similar salinity range (0–4 PSU) [97]. As suggested by Ameryk et al. (2021), this positive correlation likely reflects the passive inflow of marine SAR11 lineages with seawater intrusion rather than in situ proliferation. Conversely, *Limnohabitans* (*Betaproteobacteria*, *Burkholderiales*) decreased in relative abundance with increasing salinity in our study (Fig. S7). This finding is also in agreement with Ameryk et al. (2021) [97], who reported that the abundance of *Limnohabitans* clades B, C, and D were negatively correlated with salinity in the Vistula Lagoon.

Our findings show that salinity shapes the diversity and composition of both prokaryotic and eukaryotic communities in the tidal reach of the Pearl River Estuary, and further influences network stability and complexity. For example, salinity-driven changes in the relative abundance of phytoplankton groups (e.g., *Dinophyta*, *Chlorophyta*) may alter the production of organic matter and detritus, which in turn could affect the abundance of heterotrophic taxa such as *Ciliophora*, *Pseudomonadota*, and *Bacteroidota*. These potential trophic linkages and cross-domain associations likely contribute to the observed shifts in network complexity.

### The Network Complexity of Single-domain and Cross-domain Networks Responds Differentially to Salinity, Whereas Stability Exhibits Consistent Patterns

Despite the inherent difficulty in measuring and quantifying microbial interactions, co-occurrence network analysis has proven to be an effective tool to explore the mutual effects of microorganisms [90, 98]. The network topological parameters have been shown to be influenced by environmental factors [99, 100]. Prokaryotic co-occurrence networks were characterized by properties that indicate low stability under salinity fluctuations, while eukaryotic networks had properties that suggest higher stability (Fig. 4a, b). Compared with prokaryotes, eukaryotes possess diverse extracellular hydrolytic enzymes, which could break down different source complex organic substances, thereby enhancing persistence in tidal reach of the estuary with significant environmental fluctuations [101, 102].

Positive correlations between taxa can be explained by facilitation or mutualism, while negative correlations might mean competition [103, 104]. In our study, we found that the stability of both single-domain and cross-domain networks showed positively correlations with robustness and P/N (Fig. 5), which indicated that microbial positive interaction might enhance network stability. Under fluctuating physicochemical conditions, positive correlations can enable cooperative species to utilize a greater portion of available resources (e.g., space, light, and nutrients) [105]. Previous studies have demonstrated that diversity has a positive effect on network stability [27, 106, 107]. On the contrary, we observed a negative relationship between node number (*n*) and network stability in both prokaryotic and cross-domain networks (Fig. 5a, c). May demonstrated through mathematical modeling that in complex systems, an increase in species diversity or interaction strength can paradoxically destabilize the entire network [108]. Hu et al. (2022) found that miniature microbial communities with higher initial diversity and stronger species interactions tended to become less stable over time [109].

Key species nodes (including module hubs, connectors, and network hubs) are crucial for maintaining network stability[30]. In this study, robustness is defined as the proportion of the remaining species in the network after random node removal. Co-occurrence networks containing more key species nodes exhibit an elevated probability of losing these functionally critical nodes during random node removal. This explains the negative correlation observed between the number of keystone taxa and stability in cross-domain co-occurrence networks (Fig. 5c).

Our research uncovered that the cross-domain co-occurrence network displayed higher network complexity, while exhibiting lower network stability compared to single-domain co-occurrence networks (Figs. 3, 4). The cross-domain network, encompassing both prokaryote and eukaryote, possessed a larger number of nodes. Consequently, increased microbial diversity generated more connections, leading to higher network complexity but lower stability. This finding supports the concept that high biodiversity can diminish network stability [108, 109].

Numerous studies have revealed positive or negative effects of the increasing salinity on the microbial network complexity and stability [6, 7, 11, 28]. However, our results differ from previous studies where a linear correlation relationship between salinity and network complexity and stability. The non-linear relationship between salinity and network stability (Fig. 4) may be explained by shifting ecological processes across the tidal cycle.

During ebb tide, the inflow of river water reduces salinity while increasing the concentration of terrestrial nutrients [110]. Consistent with this general pattern, our data showed that periods of falling sea level coincided with low salinity and significantly elevated levels of turbidity and dissolved nitrogen (DN) (Fig. S4). As the previous study, the phytoplankton community is primarily dominated by *Synechococcus* and diatoms, which are known to release amino acids and polysaccharides [111, 112]. These labile substrates are rapidly metabolized by bacterioplankton, fueling dynamic carbon cycling and supporting the high uptake rates and rapid growth typical of r-strategists [110]. Correspondingly, the prokaryotic community was largely dominated by *Bacteroidota* (Fig. 2a), well-established r-strategists specialized in degrading complex organic compounds [113]. The r-strategists in nutrient-enriched environments has been shown to directly promote stronger competitive interactions among bacteria [110]. We observed that during this phase, the positive-to-negative edge ratio (P/N) in co-occurrence networks decreased, suggesting an increase in competitive (negative) interactions (Fig. 3). Allesina et al. (2012) have established that competitive interactions are inherently destabilizing in complex ecological networks [114]. Therefore, the initial decline in network stability along the salinity gradient in our study is consistent with the principle that an increase in competitive pressure, driven by terrestrial nutrient input, can compromise the overall stability of the microbial community.

During the flood tide, characterized by the inflow of seawater, the salinity of the estuary increases while nutrient concentrations tend to decrease compared to ebb tide [5, 115]. Elevated salinity exerted inhibitory effects on microbial growth, resulting in the loss of the biodiversity (Fig. 1b, e). The decline in biodiversity at high salinity levels may exacerbate the reduction in microbial function and activity. Previous studies found that higher salinity reduce microbial metabolic activities, such as phosphatase and N-acetylglucosaminidase activity [116]. Additionally, the elevated salinity can affect the osmotic pressure of microbial cells, resulting in the elimination of strains that are not adapted to high salinity [20]. When nutrient concentrations are low, microbes may cooperate to utilize organic matter in order to survive under environmental stress [117–119], leading to the formation of mutualistic relationships [120]. We did find correlations (edges) linking OTUs/ASVs classified under *Dinophyta* and *Bacteroidota*. *Bacteroidota* are often among the common bacterial taxa found in the phycosphere of dinoflagellates. *Dinoflagellates* frequently engage in symbiotic relationships with heterotrophic bacteria, including *Bacteroidota*, in marine environments [121, 122]. We speculate that *Bacteroidota* may specialize in degrading dinophyte-derived organic compounds or in providing essential micronutrients (e.g., vitamin B12, DMSP) to dinophytes [122]. Thus, higher salinity enhanced microbial cooperation and increased network stability in medium and high salinity range (1.81–6.68 PSU) (Fig. 4).

Therefore, at low salinity, network stability decreases with increasing salinity, while at high salinity, network stability increases with salinity. Overall, within a complete tidal cycle, the stability of the microbial co-occurrence networks in the tidal reach of the Pearl River Estuary exhibits a non-linear relationship, initially decreasing and then increasing with salinity fluctuations (Fig. 4).

### Limitation

This study has several limitations. First, our sampling covered a single 24-h tidal cycle, limiting statistical power and generalizability. Salinity and tidal cycle are inherently collinear in this design, preventing us from isolating salinity effects from other time-correlated factors (e.g., sunlight). The observed patterns should thus be considered hypothesis-generating, warranting validation through replicated time series or experimental manipulations.

Second, methodological constraints exist. The 27F/1492R primers do not efficiently capture Archaea, and light availability was not measured—both factors worth addressing in future work.

Third, our co-occurrence networks represent statistical associations, not direct evidence of ecological interactions. Additionally, relative abundance data cannot distinguish absolute increases from apparent gains due to declines in other taxa. Future studies should combine absolute quantification (e.g., qPCR) with cultivation-based approaches to validate the inferred interactions.

## Conclusion

In this study, we elucidated the influence of periodic salinity fluctuations on microbial diversity, community composition, and co-occurrence network topological parameters in the tidal reach of the PRE. Salinity emerged as the primary environmental factor influencing on the microbial community. As the salinity increased, the diversity of both prokaryote and eukaryote decreased. Furthermore, we found that microbial network complexity responded differently to periodic salinity fluctuations in the tidal reach, whereas the network stability showed similar pattern. Due to tidal dynamics, both single- and cross-domain network complexity and network stability displayed a non-linear relationship as the elevated salinity due to the tidal change. Overall, this study provided more comprehensive knowledge about the effect periodic salinity fluctuations on the microbial diversity, community composition, and network properties, advancing our understanding of the estuarine ecosystem.

## Supplementary Information

Below is the link to the electronic supplementary material.

**Fig. S1** Location map of the study site in the Yamen Outlet, Huangmao Sea, China. The red point shows the sampling site.

**Fig. S2** Physicochemical properties of 24 water samples in tidal reach of the PRE.

**Fig. S3** Three indices of alpha diversity in free-living and particle-attached community** (a)**. The distance-based redundancy analysis (db-RDA) results of free-living **(b)** and particle-attached **(c)** community.

**Fig. S4** Hierarchical clustering of the Bray-Curtis dissimilarity among 24 samples in prokaryotic **(a)** and eukaryotic **(b)** communites aligned with the relative abundance of different phyla. Top 10 abundant phyla were showed and the rest were incorporated and exhibited as “Others”.

**Fig. S5** Variation of Shannon entropy and reverse Simpson index of 24 samples over time in prokaryotic** (a, c)** and eukaryotic **(e, g) **communities. Relationships between salinity, Shannon entropy, and reversed Simpson index of prokaryotic **(b, d)** and eukaryotic **(f, h) **communities. The adjusted R2 and P values are shown.

**Fig. S6** Relationship between salinity and the relative abundance of the top 9 phyla in the prokaryotic **(a)** and eukaryotic **(b)** community composition among 24 samples. All remaining phyla were grouped as "Others".

**Fig. S7** Relationship between salinity and the relative abundance of the top 9 orders in the prokaryotic** (a)** and eukaryotic** (b)** community composition among 24 samples. All remaining orders were grouped as "Others".

**Fig. S8** Correlations between environmental variables and microbial diversity **(a)**, and microbial community **(b)**. Edge width corresponds to the absolute value of the correlation coefficient determined by the linear mixed-effects models. Colors indicate correlation types. *, ** and *** indicate the significance level at *P* < 0.05, *P* < 0.01 and *P* < 0.001. Temp, temperature.

**Fig. S9** Principal coordinates analysis (PCoA) of prokaryotic **(a)** and eukaryotic **(b)** community structures based on Bray-Curtis distance.

**Fig. S10** The relative abundances of key species in the prokaryotic **(a)**, eukaryotic **(b)** and multi-domain **(c)** network community composition among 24 water samples. Top 10 abundant phyla are showed and the rest were incorporated and exhibited as “Others”. High, high salinity; Medium, medium salinity; Low, low salinity.

**Fig. S11** Relationships between turbidity **(a)**, cell concentration **(b)**, and species richness of prokaryotic and eukaryotic communities.

**Fig. S12** Relationship of network complexity and stability with cell concentration.** (a) **prokaryote, **(b) **eukaryote, **(c)** cross-domain (prokaryote + eukaryote).

**Fig. S13** Relationship of network complexity and stability with turbidity. **(a)** prokaryote,** (b)** eukaryote, **(c)** cross-domain (prokaryote + eukaryote).

**Table S1** Prokaryotic co-occurrence network edge list

**Table S2 **Eukaryotic co-occurrence network edge list

**Table S3** Cross-domain (prokaryote + eukaryote) co-occurrence network edge list

## Data Availability

All sequencing data were deposited in the National Center for Biotechnology Information (https://www.ncbi.nlm.nih.gov/) under the accession number of PRJNA1310827 (16S rRNA genes) and PRJNA1310809 (ITS region).
