# Supplementary material for: Third-generation RNA Amplicon Sequencing Reveals the Dynamics of Microbial Communities in the Tidal Reach of a Subtropical Estuary
Source: Microb Ecol. 2026 Jun 23;89:172. doi: 10.1007/s00248-026-02804-4 (PMC13550336; doi:10.1007/s00248-026-02804-4)
Supplement: Supplementary file 1 [file 248_2026_2804_MOESM10_ESM.pdf]

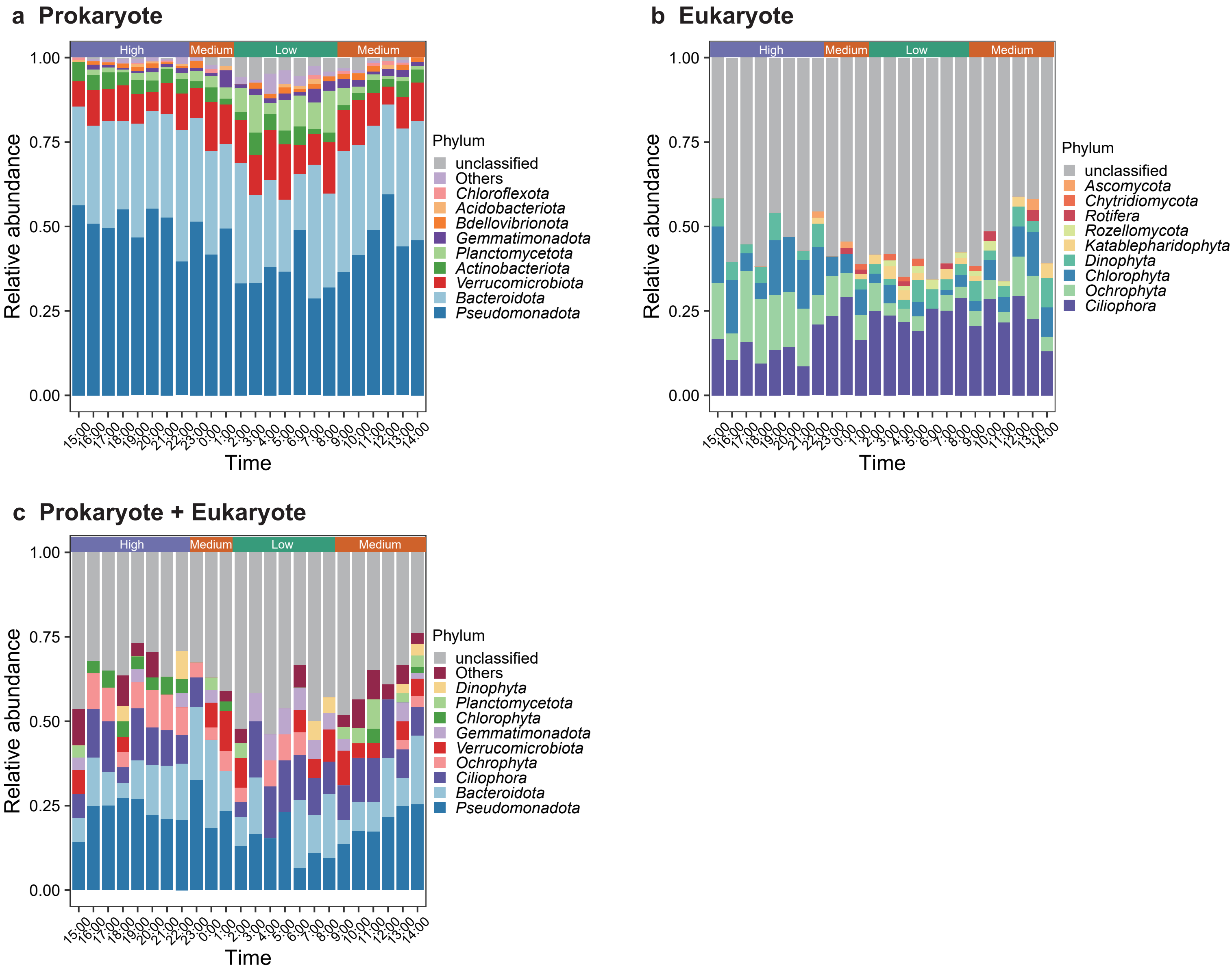
a Prokaryote
High
Medium
Low
Medium
Relative abundance
1.00
0.75
0.50
0.25
0.00
Time
15:00
16:00
17:00
18:00
19:00
20:00
21:00
22:00
23:00
0:00
1:00
2:00
3:00
4:00
5:00
6:00
7:00
8:00
9:00
10:00
11:00
12:00
13:00
14:00
Phylum
unclassified
Others
Chloroflexota
Acidobacteriota
Bdellovibrionota
Gemmatimonadota
Planctomycetota
Actinobacteriota
Verrucomicrobiota
Bacteroidota
Pseudomonadota
b Eukaryote
High
Medium
Low
Medium
Relative abundance
Time
Phylum
unclassified
Ascomycota
Chytridiomycota
Rotifera
Rozellomycota
Katablepharidophyta
Dinophyta
Chlorophyta
Ochrophyta
Ciliophora
c Prokaryote + Eukaryote
High
Medium
Low
Medium
Relative abundance
Time
Phylum
unclassified
Others
Dinophyta
Planctomycetota
Chlorophyta
Gemmatimonadota
Verrucomicrobiota
Ochrophyta
Ciliophora
Bacteroidota
Pseudomonadota
