# Supplementary material for: Third-generation RNA Amplicon Sequencing Reveals the Dynamics of Microbial Communities in the Tidal Reach of a Subtropical Estuary
Source: Microb Ecol. 2026 Jun 23;89:172. doi: 10.1007/s00248-026-02804-4 (PMC13550336; doi:10.1007/s00248-026-02804-4)
Supplement: Supplementary file 2 [file 248_2026_2804_MOESM11_ESM.pdf]

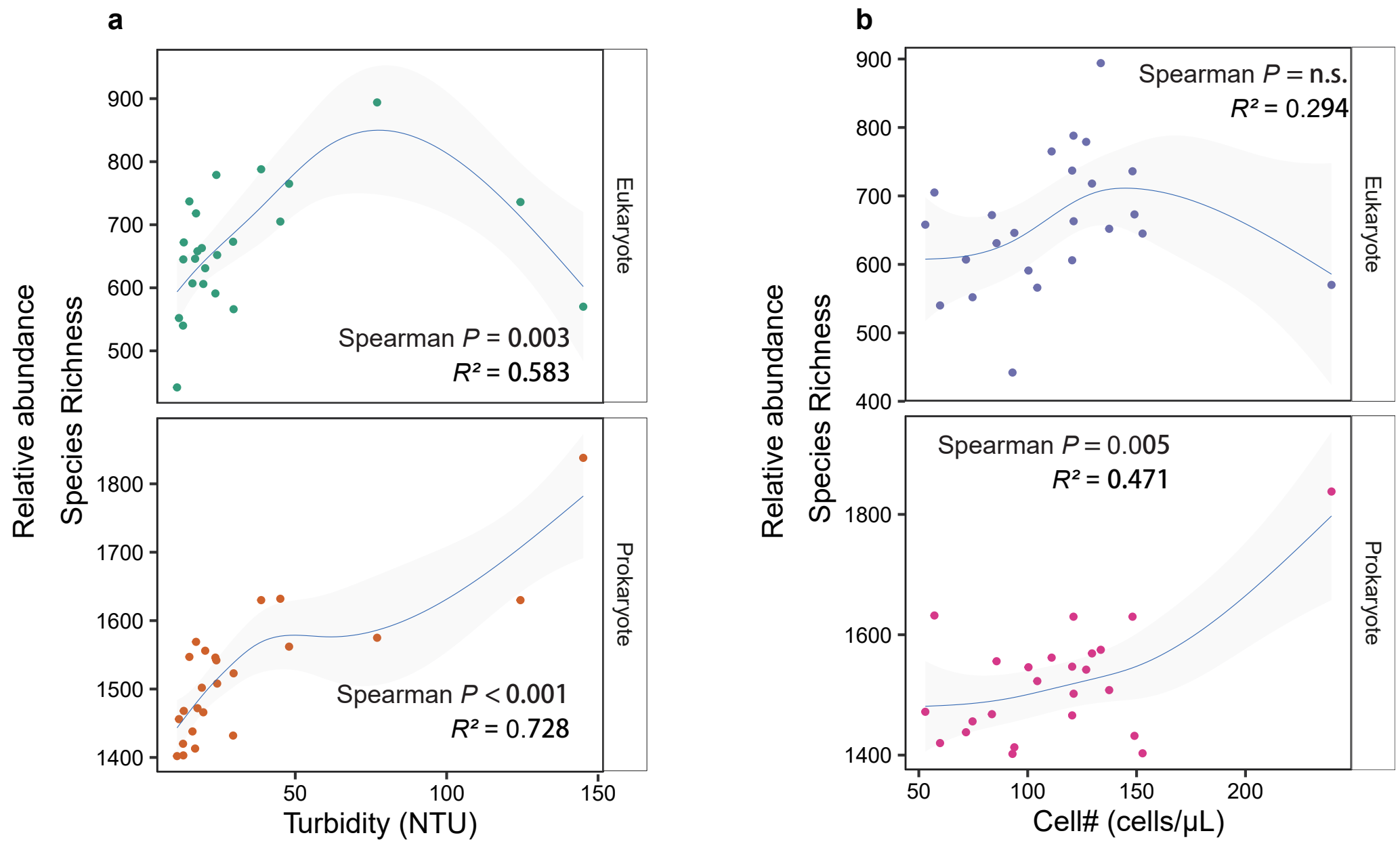

a
Relative abundance
Species Richness
900
800
700
600
500
Spearman P = 0.003
R² = 0.583
Eukaryote
1800
1700
1600
1500
1400
Spearman P < 0.001
R² = 0.728
Prokaryote
50
100
150
Turbidity (NTU)
b
Relative abundance
Species Richness
900
800
700
600
500
400
Spearman P = n.s.
R² = 0.294
Eukaryote
1800
1600
1400
Spearman P = 0.005
R² = 0.471
Prokaryote
50
100
150
200
Cell# (cells/μL)
