# Supplementary material for: Third-generation RNA Amplicon Sequencing Reveals the Dynamics of Microbial Communities in the Tidal Reach of a Subtropical Estuary
Source: Microb Ecol. 2026 Jun 23;89:172. doi: 10.1007/s00248-026-02804-4 (PMC13550336; doi:10.1007/s00248-026-02804-4)
Supplement: Supplementary file 3 [file 248_2026_2804_MOESM12_ESM.pdf]

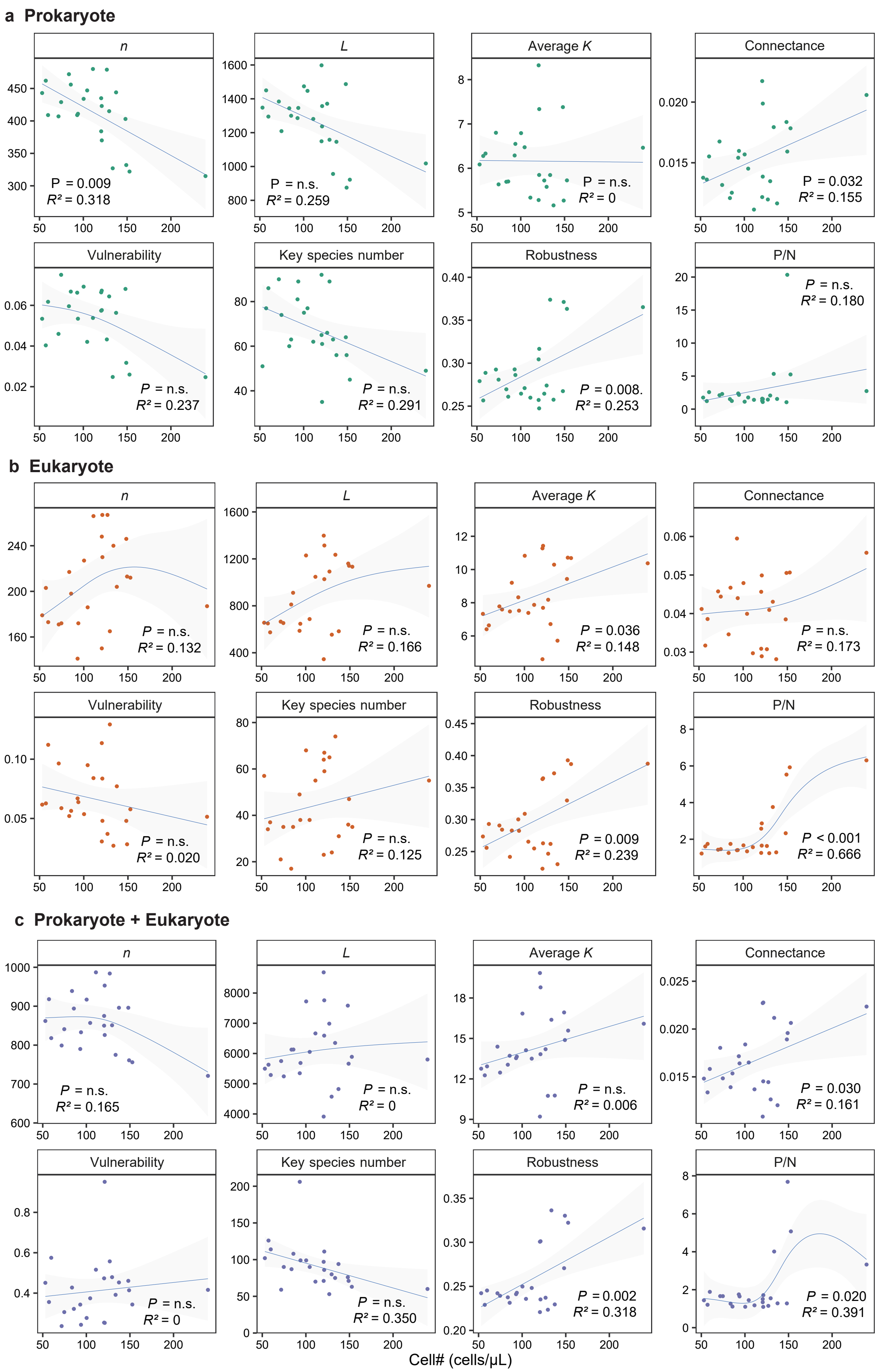

a Prokaryote
n
P = 0.009
R² = 0.318
L
P = n.s.
R² = 0.259
Average K
P = n.s.
R² = 0
Connectance
P = 0.032
R² = 0.155
Vulnerability
P = n.s.
R² = 0.237
Key species number
P = n.s.
R² = 0.291
Robustness
P = 0.008.
R² = 0.253
P/N
P = n.s.
R² = 0.180
b Eukaryote
n
P = n.s.
R² = 0.132
L
P = n.s.
R² = 0.166
Average K
P = 0.036
R² = 0.148
Connectance
P = n.s.
R² = 0.173
Vulnerability
P = n.s.
R² = 0.020
Key species number
P = n.s.
R² = 0.125
Robustness
P = 0.009
R² = 0.239
P/N
P < 0.001
R² = 0.666
c Prokaryote + Eukaryote
n
P = n.s.
R² = 0.165
L
P = n.s.
R² = 0
Average K
P = n.s.
R² = 0.006
Connectance
P = 0.030
R² = 0.161
Vulnerability
P = n.s.
R² = 0
Key species number
P = n.s.
R² = 0.350
Robustness
P = 0.002
R² = 0.318
P/N
P = 0.020
R² = 0.391
Cell# (cells/μL)
