# Supplementary material for: Third-generation RNA Amplicon Sequencing Reveals the Dynamics of Microbial Communities in the Tidal Reach of a Subtropical Estuary
Source: Microb Ecol. 2026 Jun 23;89:172. doi: 10.1007/s00248-026-02804-4 (PMC13550336; doi:10.1007/s00248-026-02804-4)
Supplement: Supplementary file 4 [file 248_2026_2804_MOESM13_ESM.pdf]

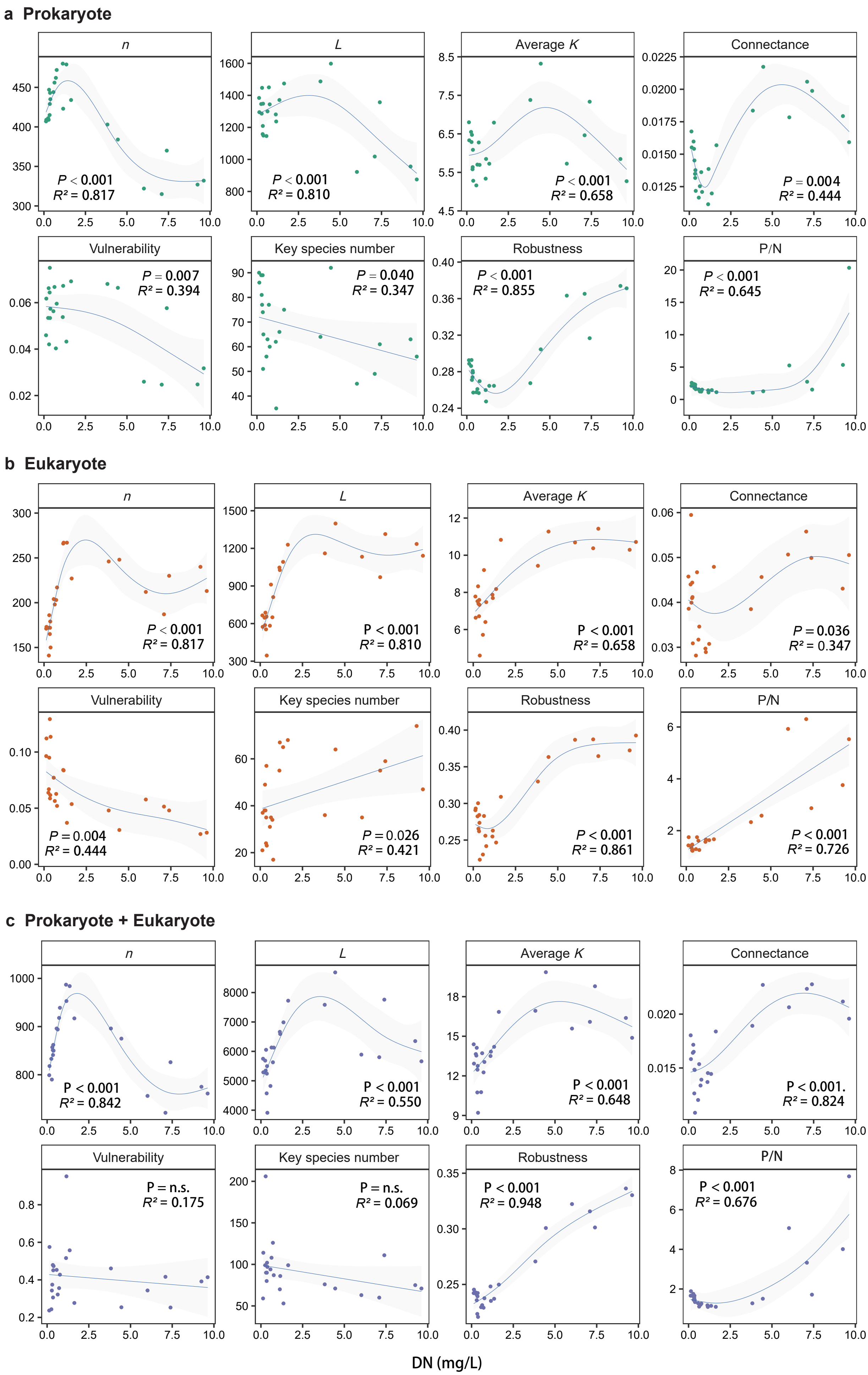
a Prokaryote
n
P < 0.001
R² = 0.817
L
P < 0.001
R² = 0.810
Average K
P < 0.001
R² = 0.658
Connectance
P = 0.004
R² = 0.444
Vulnerability
P = 0.007
R² = 0.394
Key species number
P = 0.040
R² = 0.347
Robustness
P < 0.001
R² = 0.855
P/N
P < 0.001
R² = 0.645
b Eukaryote
n
P < 0.001
R² = 0.817
L
P < 0.001
R² = 0.810
Average K
P < 0.001
R² = 0.658
Connectance
P = 0.036
R² = 0.347
Vulnerability
P = 0.004
R² = 0.444
Key species number
P = 0.026
R² = 0.421
Robustness
P < 0.001
R² = 0.861
P/N
P < 0.001
R² = 0.726
c Prokaryote + Eukaryote
n
P < 0.001
R² = 0.842
L
P < 0.001
R² = 0.550
Average K
P < 0.001
R² = 0.648
Connectance
P < 0.001.
R² = 0.824
Vulnerability
P = n.s.
R² = 0.175
Key species number
P = n.s.
R² = 0.069
Robustness
P < 0.001
R² = 0.948
P/N
P < 0.001
R² = 0.676
DN (mg/L)
