# Supplementary material for: Third-generation RNA Amplicon Sequencing Reveals the Dynamics of Microbial Communities in the Tidal Reach of a Subtropical Estuary
Source: Microb Ecol. 2026 Jun 23;89:172. doi: 10.1007/s00248-026-02804-4 (PMC13550336; doi:10.1007/s00248-026-02804-4)
Supplement: Supplementary file 5 [file 248_2026_2804_MOESM1_ESM.pdf]

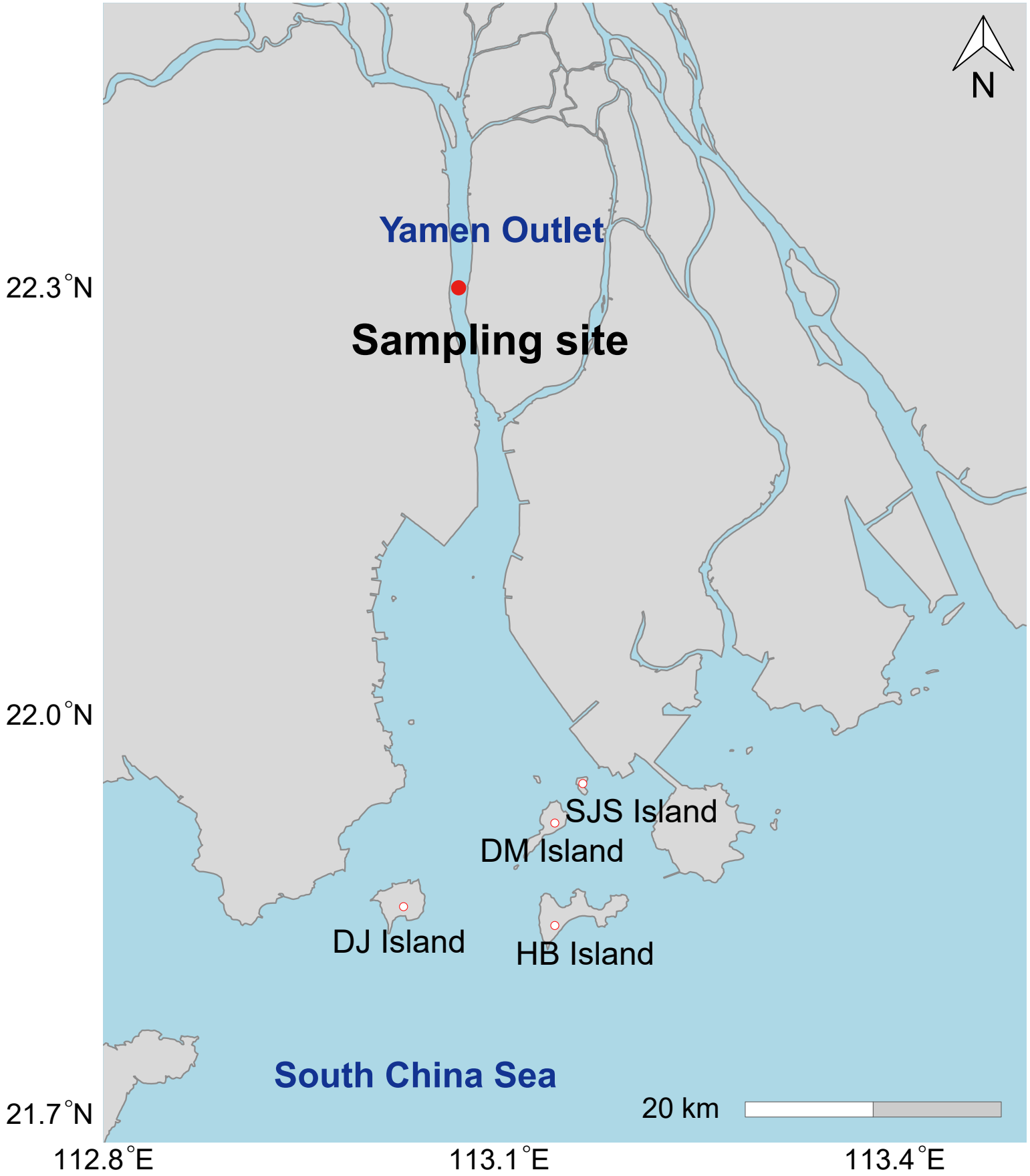

N
Yamen Outlet
Sampling site
22.3°N
22.0°N
21.7°N
SJS Island
DM Island
DJ Island
HB Island
South China Sea
20 km
112.8°E
113.1°E
113.4°E
