# Supplementary material for: Third-generation RNA Amplicon Sequencing Reveals the Dynamics of Microbial Communities in the Tidal Reach of a Subtropical Estuary
Source: Microb Ecol. 2026 Jun 23;89:172. doi: 10.1007/s00248-026-02804-4 (PMC13550336; doi:10.1007/s00248-026-02804-4)
Supplement: Supplementary file 6 [file 248_2026_2804_MOESM2_ESM.pdf]

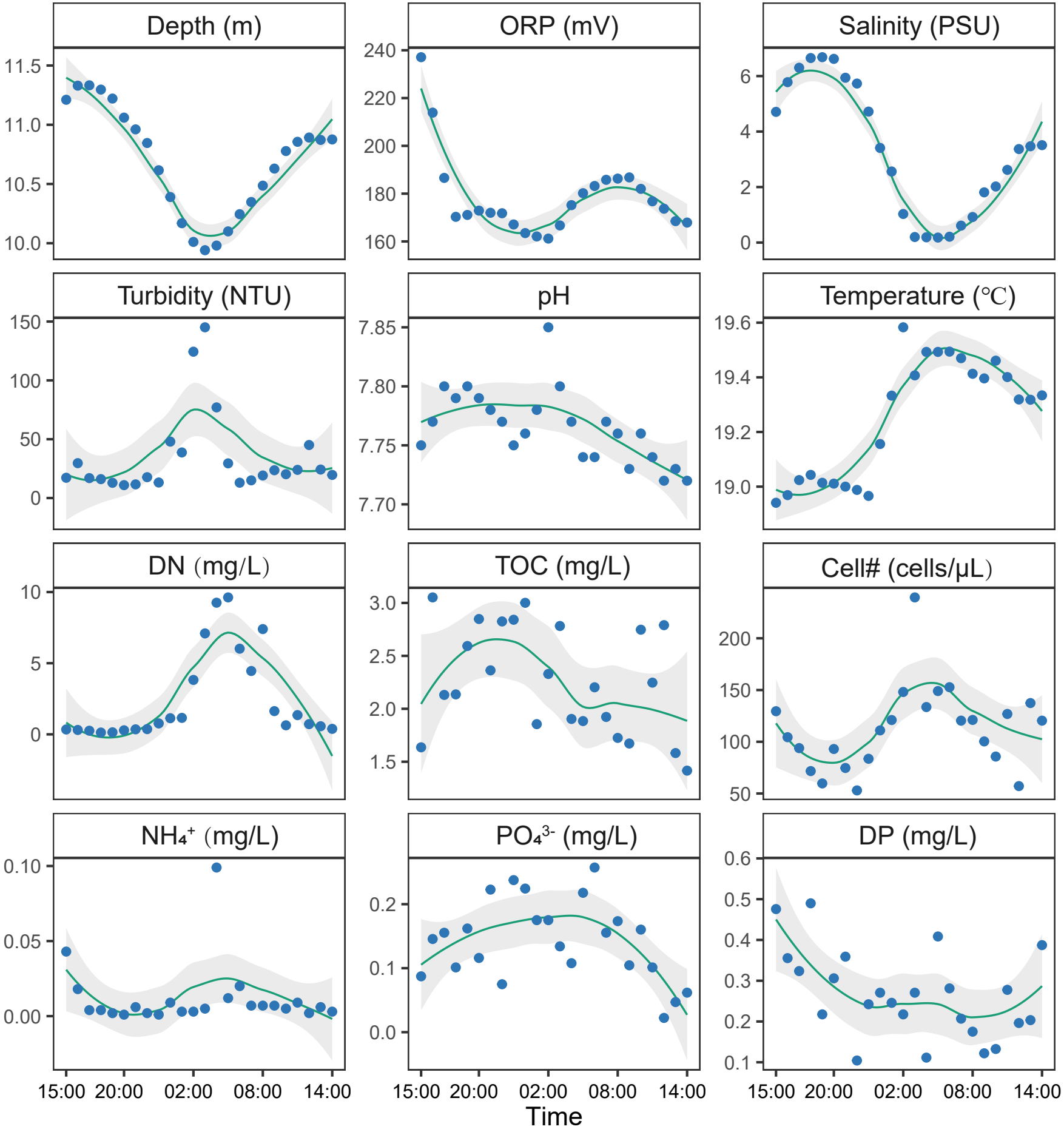

Depth (m)
ORP (mV)
Salinity (PSU)
Turbidity (NTU)
pH
Temperature (℃)
DN (mg/L)
TOC (mg/L)
Cell# (cells/μL)
NH₄⁺ (mg/L)
PO₄³⁻ (mg/L)
DP (mg/L)
15:00
20:00
02:00
08:00
14:00
Time
