# Supplementary material for: Third-generation RNA Amplicon Sequencing Reveals the Dynamics of Microbial Communities in the Tidal Reach of a Subtropical Estuary
Source: Microb Ecol. 2026 Jun 23;89:172. doi: 10.1007/s00248-026-02804-4 (PMC13550336; doi:10.1007/s00248-026-02804-4)
Supplement: Supplementary file 7 [file 248_2026_2804_MOESM3_ESM.pdf]

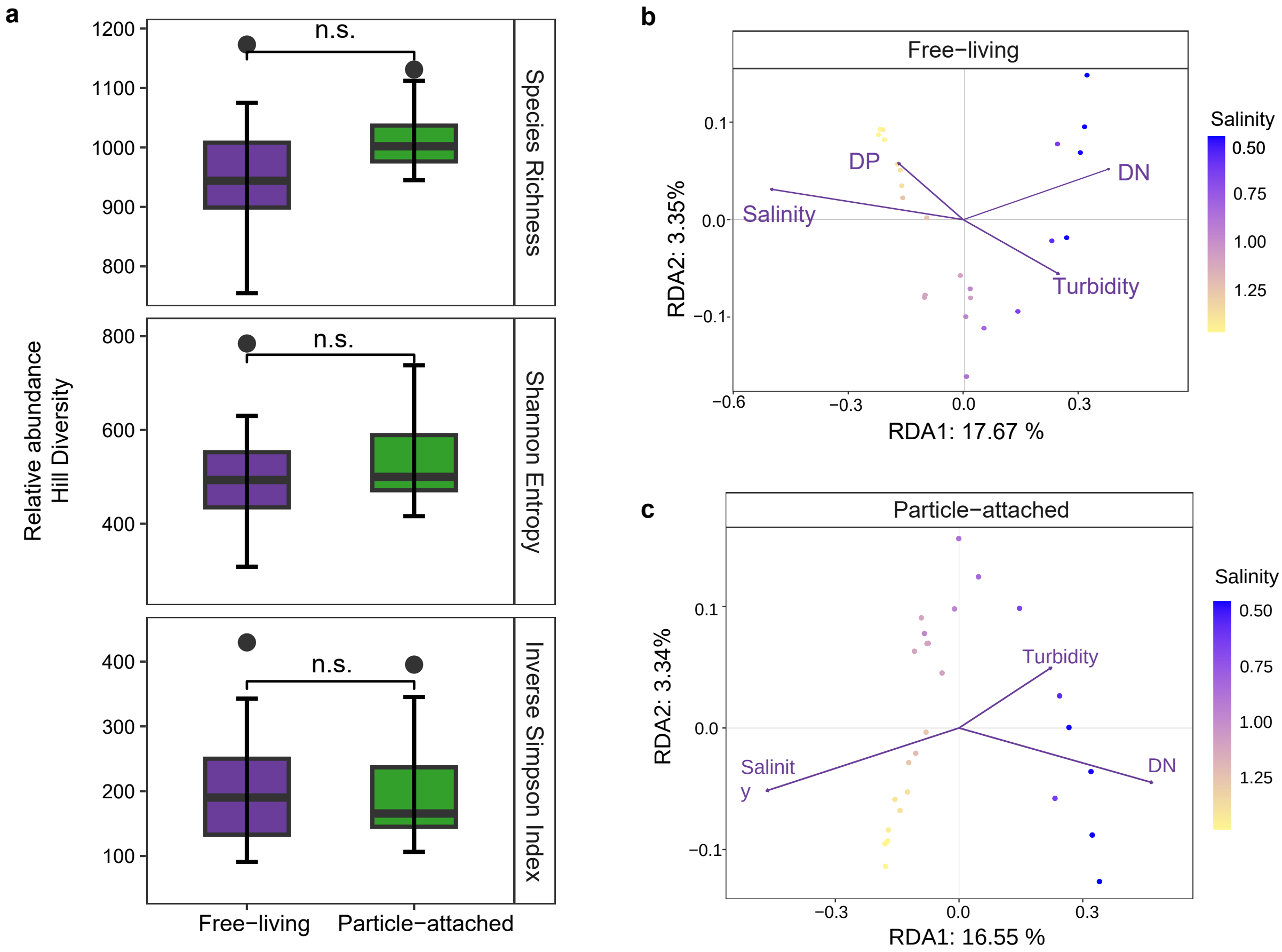
a
b
c
Relative abundance
Hill Diversity
Species Richness
Shannon Entropy
Inverse Simpson Index
n.s.
n.s.
n.s.
Free−living
Particle−attached
Free−living
Particle−attached
Salinity
DP
DN
Turbidity
Salinit
y
RDA1: 17.67 %
RDA2: 3.35%
RDA1: 16.55 %
RDA2: 3.34%
0.50
0.75
1.00
1.25
