# Supplementary material for: Third-generation RNA Amplicon Sequencing Reveals the Dynamics of Microbial Communities in the Tidal Reach of a Subtropical Estuary
Source: Microb Ecol. 2026 Jun 23;89:172. doi: 10.1007/s00248-026-02804-4 (PMC13550336; doi:10.1007/s00248-026-02804-4)
Supplement: Supplementary file 8 [file 248_2026_2804_MOESM4_ESM.pdf]

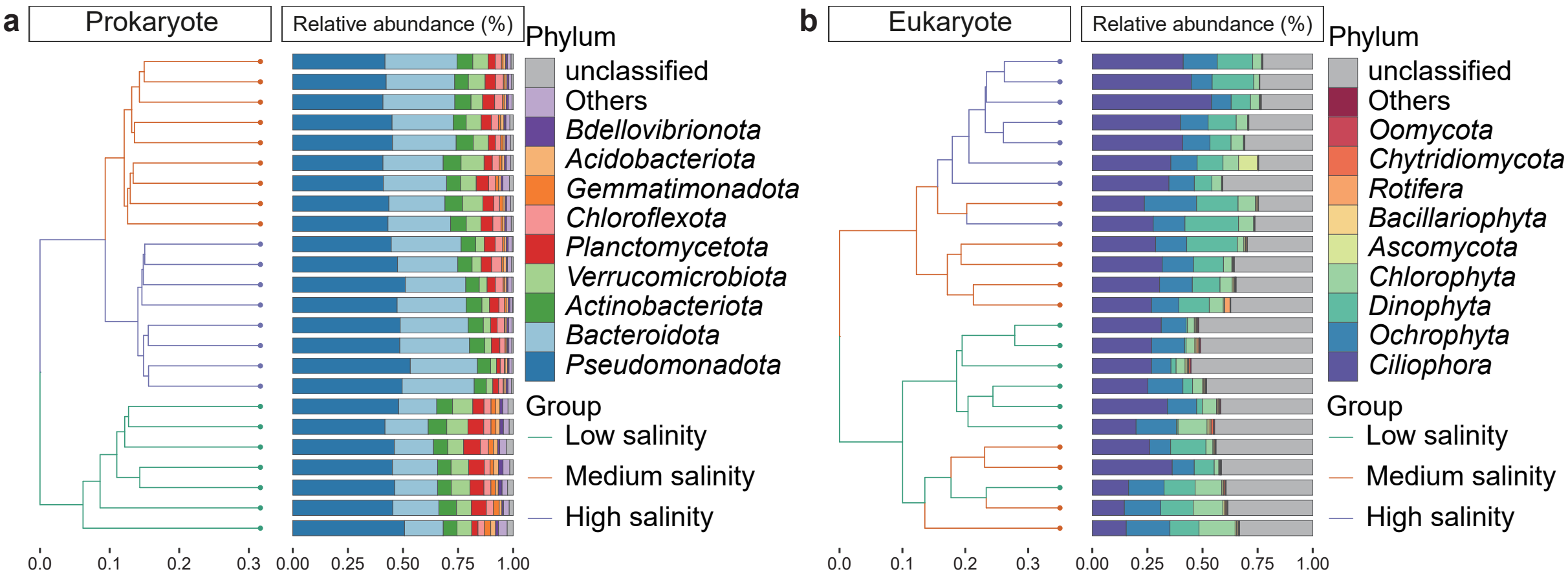
a
Prokaryote
Relative abundance (%)
Phylum
unclassified
Others
Bdellovibrionota
Acidobacteriota
Gemmatimonadota
Chloroflexota
Planctomycetota
Verrucomicrobiota
Actinobacteriota
Bacteroidota
Pseudomonadota
Group
Low salinity
Medium salinity
High salinity
0.0 0.1 0.2 0.3
0.00 0.25 0.50 0.75 1.00
b
Eukaryote
Relative abundance (%)
Phylum
unclassified
Others
Oomycota
Chytridiomycota
Rotifera
Bacillariophyta
Ascomycota
Chlorophyta
Dinophyta
Ochrophyta
Ciliophora
Group
Low salinity
Medium salinity
High salinity
0.0 0.1 0.2 0.3
0.00 0.25 0.50 0.75 1.00
