# Supplementary material for: Third-generation RNA Amplicon Sequencing Reveals the Dynamics of Microbial Communities in the Tidal Reach of a Subtropical Estuary
Source: Microb Ecol. 2026 Jun 23;89:172. doi: 10.1007/s00248-026-02804-4 (PMC13550336; doi:10.1007/s00248-026-02804-4)
Supplement: Supplementary file 9 [file 248_2026_2804_MOESM5_ESM.pdf]

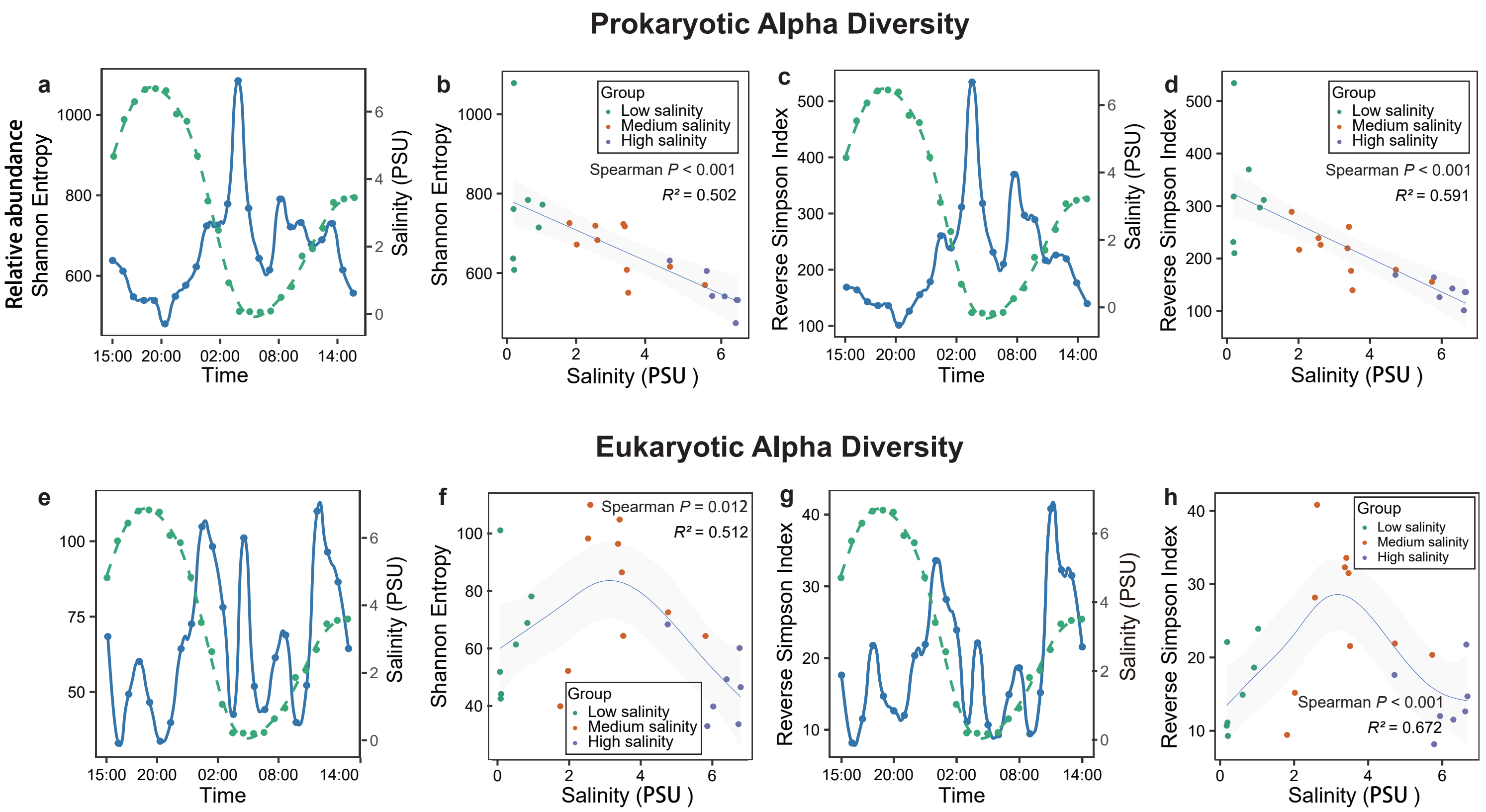
Prokaryotic Alpha Diversity
a
Relative abundance
Shannon Entropy
Salinity (PSU)
Time
b
Shannon Entropy
Group
Low salinity
Medium salinity
High salinity
Spearman P < 0.001
R² = 0.502
Salinity (PSU)
c
Reverse Simpson Index
Salinity (PSU)
Time
d
Reverse Simpson Index
Group
Low salinity
Medium salinity
High salinity
Spearman P < 0.001
R² = 0.591
Salinity (PSU)
Eukaryotic Alpha Diversity
e
Salinity (PSU)
Time
f
Shannon Entropy
Spearman P = 0.012
R² = 0.512
Group
Low salinity
Medium salinity
High salinity
Salinity (PSU)
g
Reverse Simpson Index
Salinity (PSU)
Time
h
Reverse Simpson Index
Group
Low salinity
Medium salinity
High salinity
Spearman P < 0.001
R² = 0.672
Salinity (PSU)
