# Supplementary material for: Third-generation RNA Amplicon Sequencing Reveals the Dynamics of Microbial Communities in the Tidal Reach of a Subtropical Estuary
Source: Microb Ecol. 2026 Jun 23;89:172. doi: 10.1007/s00248-026-02804-4 (PMC13550336; doi:10.1007/s00248-026-02804-4)
Supplement: Supplementary file 10 [file 248_2026_2804_MOESM6_ESM.pdf]

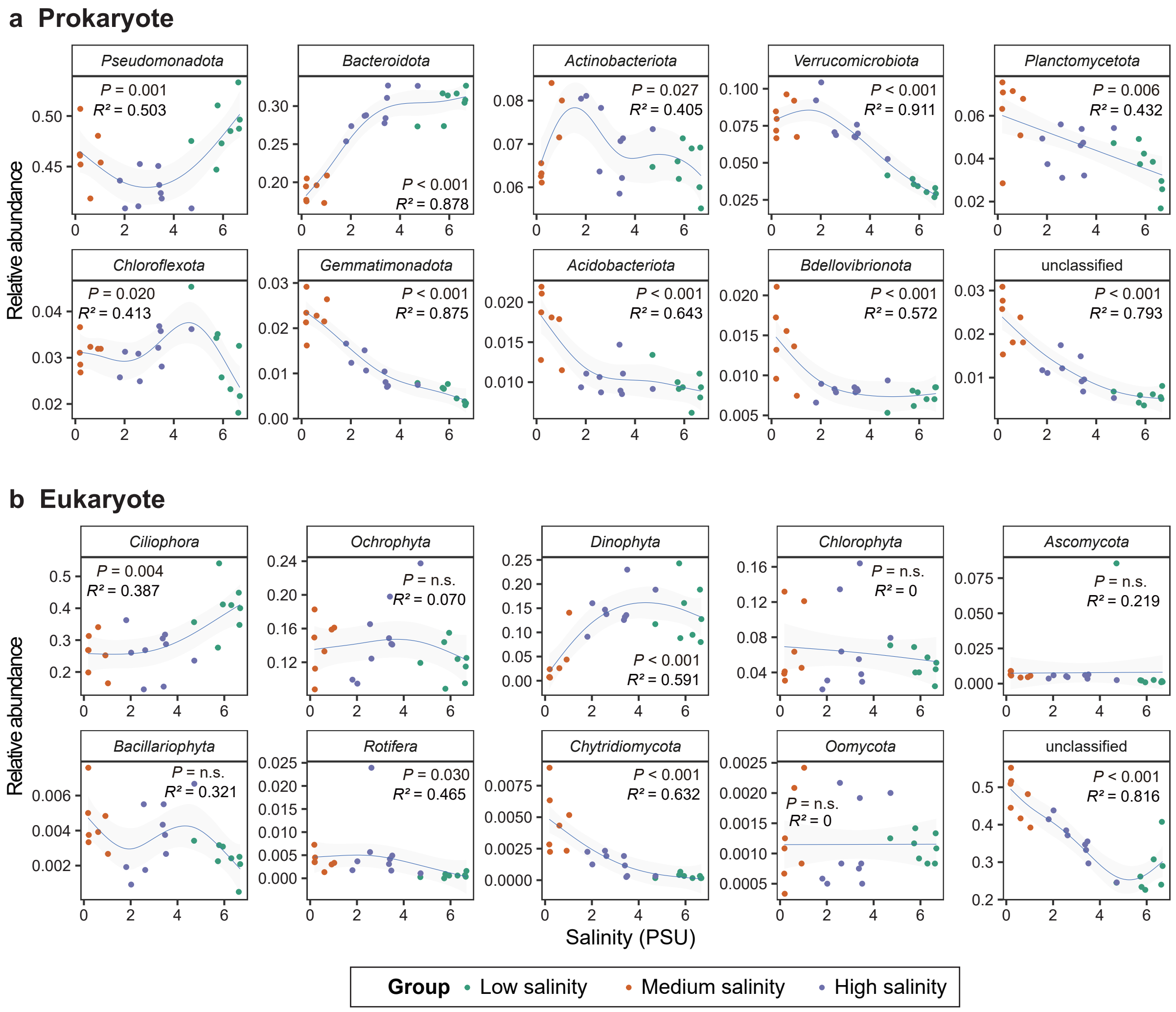
a Prokaryote
Pseudomonadota
P = 0.001
R² = 0.503
Bacteroidota
P < 0.001
R² = 0.878
Actinobacteriota
P = 0.027
R² = 0.405
Verrucomicrobiota
P < 0.001
R² = 0.911
Planctomycetota
P = 0.006
R² = 0.432
Chloroflexota
P = 0.020
R² = 0.413
Gemmatimonadota
P < 0.001
R² = 0.875
Acidobacteriota
P < 0.001
R² = 0.643
Bdellovibrionota
P < 0.001
R² = 0.572
unclassified
P < 0.001
R² = 0.793
Relative abundance
b Eukaryote
Ciliophora
P = 0.004
R² = 0.387
Ochrophyta
P = n.s.
R² = 0.070
Dinophyta
P < 0.001
R² = 0.591
Chlorophyta
P = n.s.
R² = 0
Ascomycota
P = n.s.
R² = 0.219
Bacillariophyta
P = n.s.
R² = 0.321
Rotifera
P = 0.030
R² = 0.465
Chytridiomycota
P < 0.001
R² = 0.632
Oomycota
P = n.s.
R² = 0
unclassified
P < 0.001
R² = 0.816
Relative abundance
Salinity (PSU)
Group
Low salinity
Medium salinity
High salinity
