# Supplementary material for: Third-generation RNA Amplicon Sequencing Reveals the Dynamics of Microbial Communities in the Tidal Reach of a Subtropical Estuary
Source: Microb Ecol. 2026 Jun 23;89:172. doi: 10.1007/s00248-026-02804-4 (PMC13550336; doi:10.1007/s00248-026-02804-4)
Supplement: Supplementary file 11 [file 248_2026_2804_MOESM7_ESM.pdf]

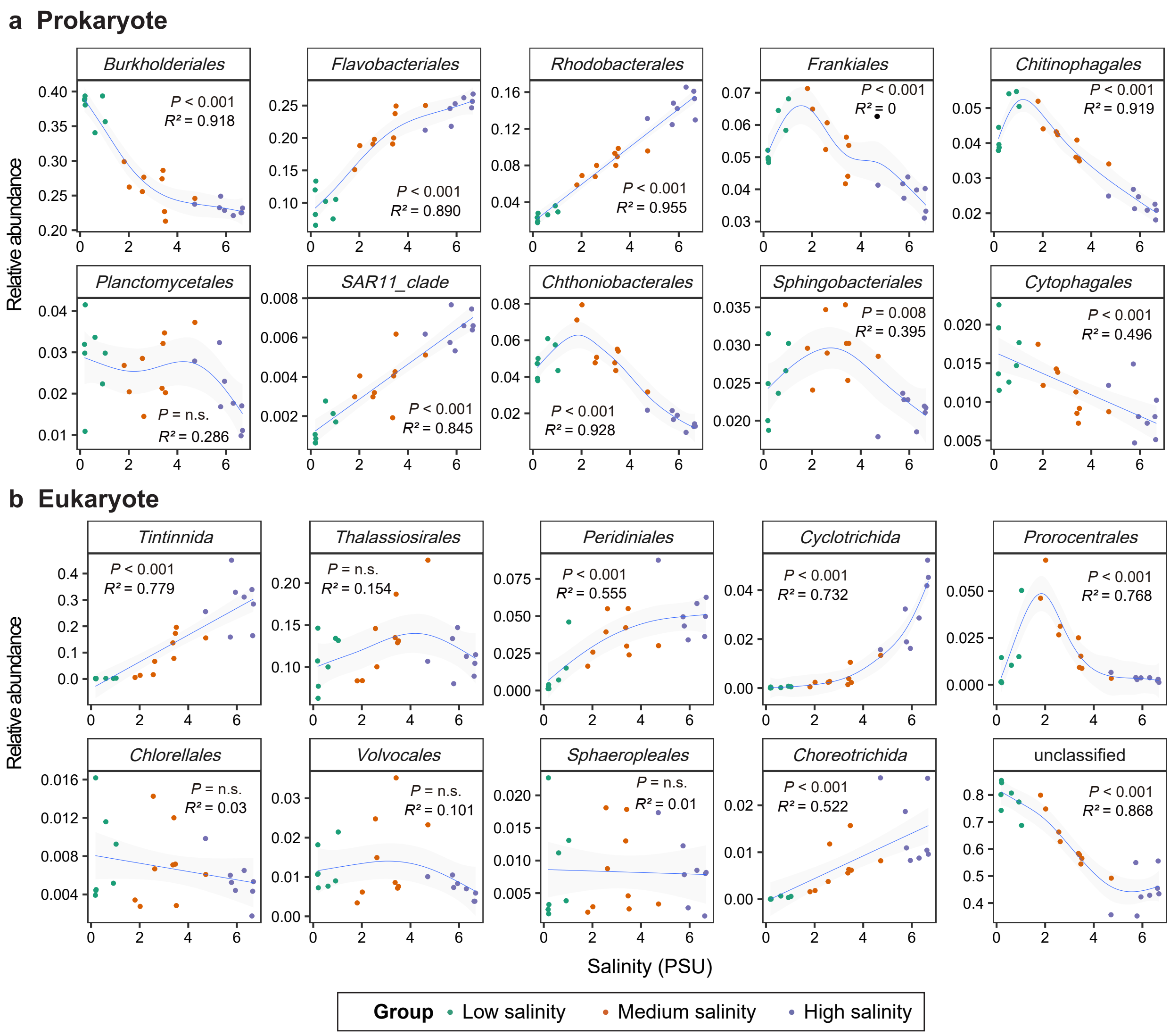

a Prokaryote
Burkholderiales
P < 0.001
R² = 0.918
Flavobacteriales
P < 0.001
R² = 0.890
Rhodobacterales
P < 0.001
R² = 0.955
Frankiales
P < 0.001
R² = 0
Chitinophagales
P < 0.001
R² = 0.919
Planctomycetales
P = n.s.
R² = 0.286
SAR11_clade
P < 0.001
R² = 0.845
Chthoniobacterales
P < 0.001
R² = 0.928
Sphingobacteriales
P = 0.008
R² = 0.395
Cytophagales
P < 0.001
R² = 0.496
Relative abundance
b Eukaryote
Tintinnida
P < 0.001
R² = 0.779
Thalassiosirales
P = n.s.
R² = 0.154
Peridiniales
P < 0.001
R² = 0.555
Cyclotrichida
P < 0.001
R² = 0.732
Prorocentrales
P < 0.001
R² = 0.768
Chlorellales
P = n.s.
R² = 0.03
Volvocales
P = n.s.
R² = 0.101
Sphaeropleales
P = n.s.
R² = 0.01
Choreotrichida
P < 0.001
R² = 0.522
unclassified
P < 0.001
R² = 0.868
Relative abundance
Salinity (PSU)
Group
Low salinity
Medium salinity
High salinity
