# Supplementary material for: Third-generation RNA Amplicon Sequencing Reveals the Dynamics of Microbial Communities in the Tidal Reach of a Subtropical Estuary
Source: Microb Ecol. 2026 Jun 23;89:172. doi: 10.1007/s00248-026-02804-4 (PMC13550336; doi:10.1007/s00248-026-02804-4)
Supplement: Supplementary file 12 [file 248_2026_2804_MOESM8_ESM.pdf]

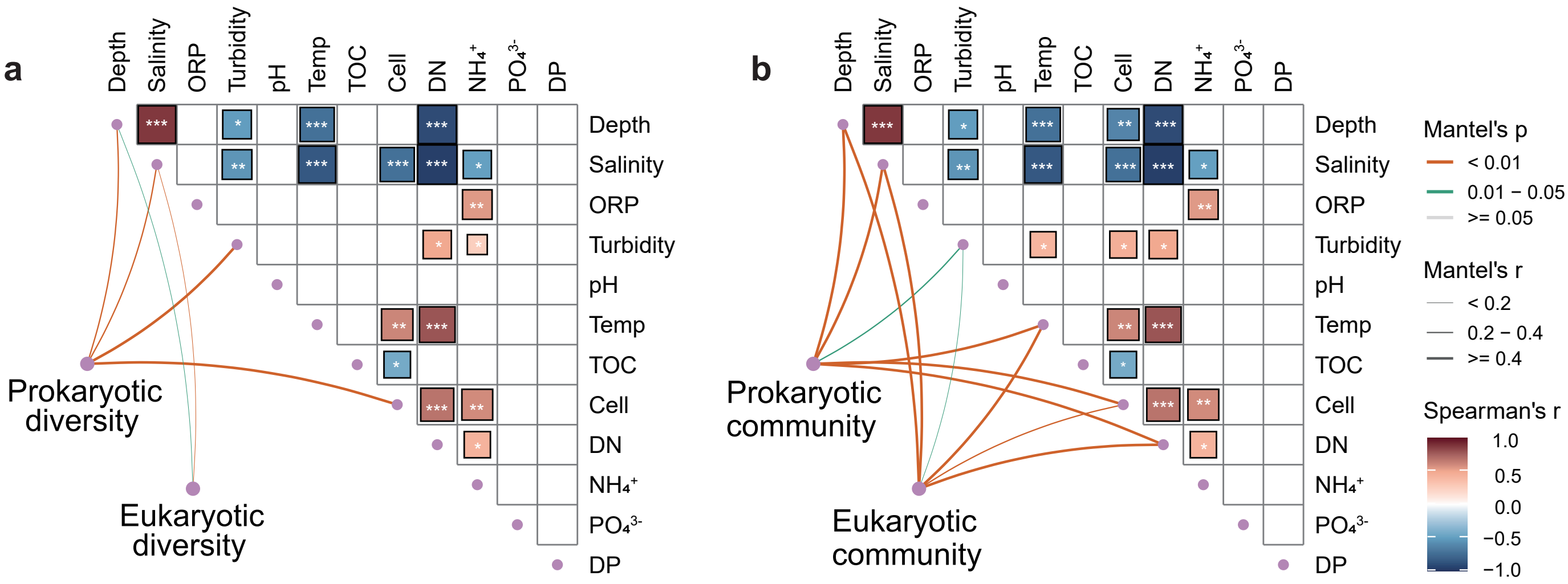
a
Depth
Salinity
ORP
Turbidity
pH
Temp
TOC
Cell
DN
NH₄⁺
PO₄³⁻
DP
Prokaryotic diversity
Eukaryotic diversity
b
Prokaryotic community
Eukaryotic community
Mantel's p
< 0.01
0.01 − 0.05
>= 0.05
Mantel's r
< 0.2
0.2 − 0.4
>= 0.4
Spearman's r
1.0
0.5
0.0
−0.5
−1.0
