# Supplementary material for: Third-generation RNA Amplicon Sequencing Reveals the Dynamics of Microbial Communities in the Tidal Reach of a Subtropical Estuary
Source: Microb Ecol. 2026 Jun 23;89:172. doi: 10.1007/s00248-026-02804-4 (PMC13550336; doi:10.1007/s00248-026-02804-4)
Supplement: Supplementary file 13 [file 248_2026_2804_MOESM9_ESM.pdf]

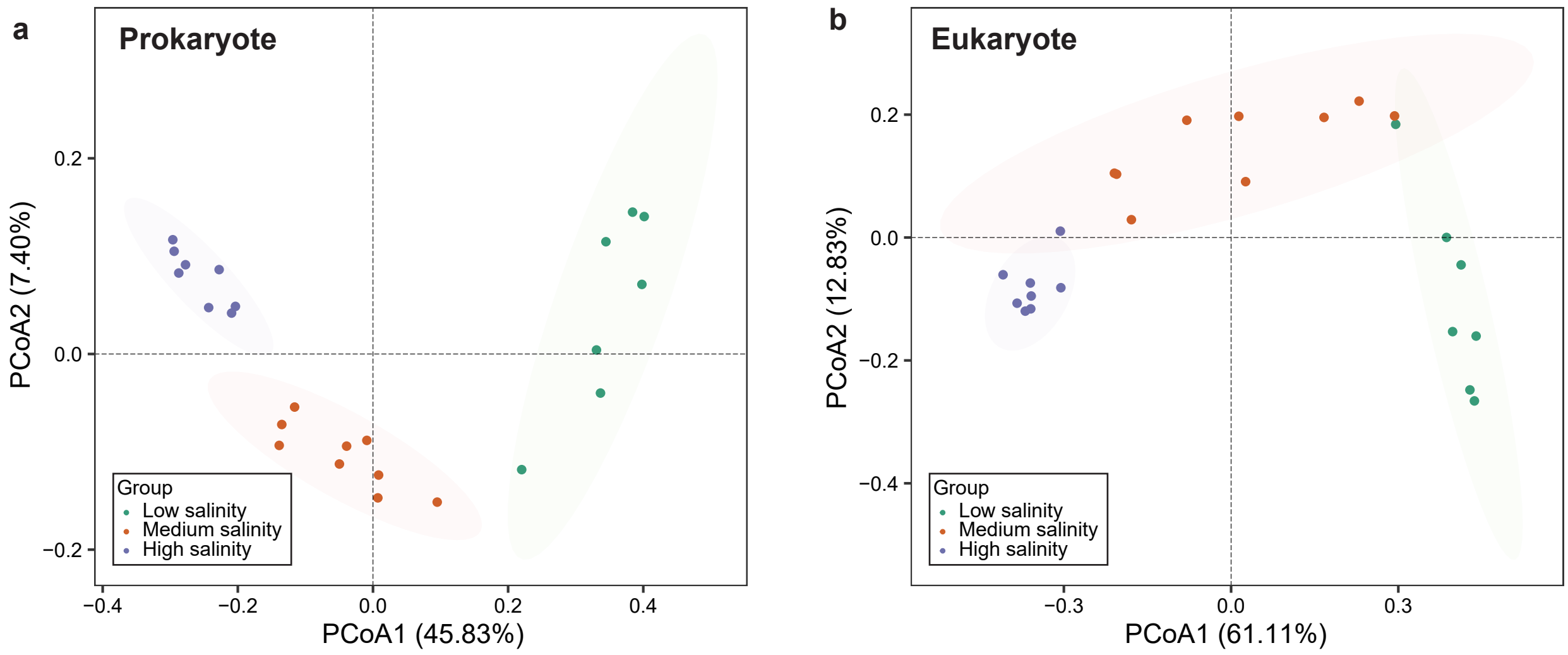
a
Prokaryote
PCoA2 (7.40%)
PCoA1 (45.83%)
0.2
0.0
−0.2
−0.4
−0.2
0.0
0.2
0.4
Group
Low salinity
Medium salinity
High salinity
b
Eukaryote
PCoA2 (12.83%)
PCoA1 (61.11%)
0.2
0.0
−0.2
−0.4
−0.3
0.0
0.3
Group
Low salinity
Medium salinity
High salinity
